# Evaluation of the Effects of Acorns on the Meat Quality and Transcriptome Profile of Finishing Yuxi Pigs

**DOI:** 10.3390/ani15050614

**Published:** 2025-02-20

**Authors:** Jinzhou Zhang, Chuankuan Zhang, Shuaitao Meng, Heming Wang, Dongyang Liu, Liping Guo, Zhiguo Miao

**Affiliations:** 1 College of Animal Science and Veterinary Medicine, Henan Institute of Science and Technology, Xinxiang 453003, China; zhangjz69@126.com (J.Z.); chuankuan0526@163.com (C.Z.); mengshuaitao2001@163.com (S.M.); sqheming@163.com (H.W.); liudy97@126.com (D.L.); 2 School of Food Science, Henan Institute of Science and Technology, Xinxiang 453003, China; lipingguo1982@126.com

**Keywords:** acorn, glycolysis, meat quality, myofiber, transcriptome, Yuxi pig

## Abstract

In this study, we explored the effects of dietary acorn on the meat quality and transcriptome profile of finishing Yuxi pigs. A total of 90 pigs (99.60 ± 1.63 kg) were randomly assigned to three groups: the control group fed a commercial diet (CN), and two treatment groups fed 100 (AC1) and 300 (AC2) g/kg of acorns, respectively. Each group contained five replicates with six pigs per replicate. After a 120-day treatment period, the AC2 group showed significantly higher pH_24h_, a*, intramuscular fat, and umami amino acid and significantly lower L*, cooking loss, and shear force than the CN group (*p* < 0.05). Further, the AC2 group showed significantly increased glycogen, ATP, and ADP, creatine kinase activity, and myofiber density and significantly decreased glycolytic potential, lactic acid, and lactate dehydrogenase, malate dehydrogenase, phosphofructokinase muscle, and pyruvate kinase activities (*p* < 0.05). The mRNA levels of *MYH7*, *MYH2*, and *MYH1* were significantly upregulated in the AC2 group (*p* < 0.05). A transcriptome analysis further revealed significant differences in gene expression patterns between the AC2 and CN groups. These findings suggest that dietary acorns at 300 g/kg improve pork quality by inducing the conversion of myofiber types and regulating glycolysis.

## Simple Summary

Acorns, the fruit of oak trees, are widely distributed throughout China. In this study, we explored the effects of dietary acorns on the meat quality and transcriptome profile of finishing Yuxi pigs, and found that dietary supplementation with 300 g/kg acorns in finishing Yuxi pigs improved pork quality by inducing myofiber conversion to types that improve pork quality and taste, regulating glycolysis, extracellular matrix formation, and substance and energy metabolism. The findings of this study highlight acorn supplementation as a strategy for improving pork quality and a basis for the development of alternative feed components enhancing the sustainability of the swine industry.

## 1. Introduction

As the scale of livestock breeding expands globally, particularly in developing countries, the demand for feed ingredients will further increase [1]. In 2011, approximately 70% of the corn produced in China was used as feed. Thus, it is expected that increasing demand for feed will continue to worsen food insecurity in several countries. Additionally, the lack of feed ingredients and increasing demand for pork represent key incompatibilities in swine production. Therefore, there is an urgent need to identify alternative natural feed resources that can serve as feed ingredients to enhance the sustainability of the swine industry [2].

Acorns, the fruit of oak trees, are widely distributed throughout China. Over 49 species of oak trees, belonging to seven varieties, cover an area greater than 16.72 million ha in China, and yield 6–7 million tons of acorns annually [3]. Acorns primarily contain starch (58%), crude protein (1.17–8.72%), crude fat (1.04–6.86%), and tannins (0.26–17.74%) [4], all of which contribute to their wide range of biological effects, including antioxidant, antimicrobial, hypolipidemic, and hypoglycemic effects [3,5]. So far, research on acorns in pigs has mainly focused on Iberian pigs. Rodríguez-Sánchez et al. [6] observed that the at the beginning of the Montanera period, which is characterized by the maturation of acorns and the availability of grass, the meat of 18-month-old Iberian pigs has higher red (a*) values and shows lower cooking loss than that of 12-month-old pigs. This observation suggests a higher pork quality for the 18-month-old group at the beginning of the Montanera period. Another study revealed no differences in carcass weight, meat composition, pH_24h_, and color of the longest lumbar muscle between Iberian pigs fed acorns and those fed a standard protein diet; however, the acorn-fed pigs showed a lower growth rate [7]. Therefore, there are inconsistencies in the results of studies on the impact of acorns on pork quality, particularly meat color and pH. In addition, some studies have investigated the net portal appearance of metabolites and amino acids in Iberian pigs fed acorns, and showed higher monounsaturated and polyunsaturated fatty acids, and lower saturated fatty acids, i.e., healthier fat, in Montanera pigs than those in intensive systems [8,9,10,11]. Our former study showed that acorns restrained subcutaneous fat deposition and improved the pork nutritional value of Yuxi pigs under extensive conditions [12].

The Yuxi pig is an indigenous Chinese breed that is primarily distributed in the mountainous regions of the western Henan Province. It is characterized by strong adaptability, high disease resistance, and high pork quality [13]. In this study, we postulated that dietary acorn supplementation exerts beneficial effects on the meat quality of Yuxi pigs. Using RNA sequencing (RNA-seq) and high-performance liquid chromatography-mass spectrometry (HPLC-MS), we assessed the effect of dietary acorns on the meat quality of the *Longissimus thoracis* muscle (LTM) of Yuxi pigs and characterized its transcriptome profile. The findings of this study may highlight acorn supplementation as a strategy for improving pork quality and also serve as a basis for the development of alternative feed components, hence enhancing the sustainability of the swine industry.

## 2. Materials and Methods

### 2.1. Animals, Trial Design, and Management

The animal trial protocol employed in this study was approved by the Animal Protection and Utilization Committee of the Henan Institute of Science and Technology, Xinxiang, China (Approval number 2020 HIST018). Ninety healthy finishing Yuxi pigs (45 castrated males and 45 females; average body weight, 99.60 ± 1.63 kg; average age, 185 ± 7 days old) were provided by Henan Heiyuan Agriculture and Animal Husbandry Technology Co., Ltd. (Zhengzhou, China). The acorns used in this study, obtained from *Quercus variabilis* Bl., which is the most common oak variety in the study area, were purchased at a local market. The nutritional contents and levels of the acorns were as follows: starch, 65.45% (Anthrone colorimetry); crude fat, 2.29% (Soxhlet extraction); crude protein, 4.41% (Kjeldahl nitrogen); and tannin, 3.11% (colorimetry). After removing the acorn shell, the kernel as well as other feed ingredients were weighed according to the feed formula provided in Table 1 and poured into a feed mill for crushing and even mixing.

The pigs were randomly divided into the CN, AC1, and AC2 groups. Those in the CN group were fed a commercial diet, while those in the two treatment groups, AC1 and AC2 were fed diets supplemented with 100 g/kg and 300 g/kg, respectively. Each group had five replicates, with six pigs per replicate (sex-balanced, a rearing pen) at a density of 3 m^2^/pig. The diets, with composition and nutritional levels as shown in Table 1, were designed in accordance with the Nutrient Requirements of Swine in China (GB/T 39235-2020) [14]. The animals were fed the different diets for 120 days at our institution and the feeding and management conditions were in accordance with piggery procedures. The pig house was maintained at a temperature of 21–25 °C and naturally ventilated, and feed was obtained at 6:00 and 18:00 twice daily. All the animals were provided with water and feed ad libitum. At the beginning and end of the treatment, the animals were fasted for 12 h but still had free access to water, and, thereafter, were weighed to determine their initial body weight (IBW) and final body weight (FBW), respectively.

### 2.2. Sample Collection

At the end of the feeding period, the animals were weighed and kept in fasting for 12 h but with ad libitum access to water. After electrical stunning, the animals were exsanguinated in a commercial abattoir.

After slaughter, pork was collected from the LTM between the 10th and 12th ribs on the left side of the carcass of 30 pigs (two pigs per replicate, i.e., one castrated male and one female) within 30 min. Next, some of the pork tissue was placed in RNAse-free tubes, rapidly frozen in liquid nitrogen, and stored at −80 °C for the subsequent RNA extraction, detection of glycolytic enzyme activity and glycolytic potential (GP), measurement of adenosine phosphate and amino acid levels, and transcriptome analyses. Another portion of the tissue was trimmed into 1-cm^3^ pieces and fixed in 4% paraformaldehyde for histological analysis. The remainder of the tissue sample was stored at 4 °C for 24 h for the evaluation of meat quality.

### 2.3. Assay of Meat Quality

The meat quality assay using LTM samples was conducted as previously described by Ortiz et al. [15], with some modifications.

#### 2.3.1. pH Values

After calibrating the pH meter at pH 7.01 and pH 4.01 and applying temperature compensation, the pH values of the LTM samples 45 min postmortem (pH_45min_) at environmental temperature and 24 h postmortem (pH_24h_) at 4 °C were determined using a portable acidimeter (PH-STAR, Matthaus, Eckelsheim, Germany).

#### 2.3.2. Meat Color

LTM samples were used to determine pork color (i.e., the lightness [L*], redness [a*], and yellowness [b*]) at 45 min at environmental temperature, and 24 h postmortem at 4 °C, after 60 min blooming at 22 °C, using a Minolta CR-400 colorimeter (Konica Minolta, Tokyo, Japan) with a D65 illuminant, 2° standard observer, and 8 mm aperture.

#### 2.3.3. Drip Loss

The LTM samples were cut into 1 × 1 × 2 cm pieces, weighed (W1), and hung in sealed plastic cups at 4 °C for 24 h. Next, the pieces were removed and weighed (W2) again after removing surface moisture using filter paper. Drip loss was then calculated according to the following equation: drip loss (%) = (W1 − W2)/W1 × 100.

#### 2.3.4. Cooking Loss

Approximately 50 g LTM samples were weighed (W3), vacuum packaged, and cooked in an 80 °C water bath until the internal temperature of the pork chunks reached 75 °C. Next, the cooked samples were left to stand in bags at 20 °C for 30 min, after which they were removed from the bags and weighed (W4) after surface moisture removal using filter paper. Thereafter, cooking loss was determined according to the following expression: cooking loss (%) = (W3 − W4)/W3 × 100.

#### 2.3.5. Shear Force

After the determination of the cooking loss, the cooked pork chunks were trimmed into 1 × 1 × 3 cm pieces (3.4 ± 0.21 g), six pieces per sample, and cut vertically along the muscle fibers at the speed of 0.83 mm/s. Then, Warner–Bratzler shear force values (N) were recorded using a muscle tenderness meter (C-LM3B, Tenovo, Beijing, China) with an electric sensor (0.05–25 kg).

### 2.4. Analysis of Chemical Composition

Moisture, protein, and ash were assessed according to the official methods [16] and expressed as a ratio relative to fresh meat weight (%).

Intramuscular fat (IMF) levels in the LTM samples were assessed via ether extraction using the Soxhlet method [17] and expressed as a ratio relative to fresh meat weight (%).

### 2.5. Analysis of Free Amino Acid Contents

LTM samples were analyzed for the contents of 18 free amino acids using an HPLC-MS (SCIEX QTRAP 4500; Applied Biosystems, Framingham, MA, USA) system coupled to an Agilent 1260 Infinity HPLC system (Agilent Technologies, Santa Clara, CA, USA) equipped with a Zorbax SB C18 column (150 × 4.6 mm, 5 μm; Agilent Technologies) as previously described by Li et al. [18], with some modifications.

### 2.6. Histological Analysis

Fixed LTM samples were sequentially dehydrated, embedded in paraffin wax, cut into 5 μm thick sections, and stained with hematoxylin and eosin. Thereafter, eight views, each containing more than 100 myofibers, were randomly chosen, photographed, and evaluated using Image-Pro Plus version 6.0 (Media Cybernetics, Rockville, MD, USA). The myofiber diameter, density, and cross-sectional area of the LTM samples were then calculated.

### 2.7. Measurement of Glycolytic Potential (GP) and Adenosine Phosphate Contents

The lactic acid and glycogen contents of the LTM samples were determined as previously described by Zhang et al. [19]. In brief, 0.5 g of frozen meat samples was added to 4.5 mL of pre-cooling HClO_4_ (0.85 mol/L) solution followed by homogenization in an ice bath and separation via centrifugation at 3500× *g* at 4 °C for 10 min. The resulting supernatant was collected, neutralized with 10 mol/L KOH, and stored at −80 °C for later use. The lactic acid and glycogen contents of the samples were then analyzed via a colorimetric assay using the respective kits (Nanjing Jiancheng Bioengineering Institute, Nanjing, China) in accordance with the manufacturer’s instructions. The results obtained were then presented as µmol/g. GP was then determined as 2 × glycogen content + lactic acid content, according to Li et al. [20].

The ATP, ADP, and AMP contents of the LTM samples were evaluated via HPLC as previously described by Wang et al. [21]. In brief, frozen meat samples (0.5 g) were homogenized in 3 mL of pre-cooling 7% HClO_4_ and centrifuged at 13,000× *g* at 4 °C for 10 min. Next, the resulting supernatant was neutralized with 0.85 mol/L KOH, and recentrifuged at 13,000× *g* for 10 min at 4 °C to remove KClO_4_. After filtration through a 0.22 μm membrane, 10 μL of the filtered solution was pumped into an Agilent 1260 Infinity HPLC system (Agilent Technologies) fitted with an Agilent Zorbax SB C18 analytical column (150 × 2.1 mm, 5 μm; Agilent Technologies) at 4 °C for analysis. The mobile phase was 0.05 mol/L phosphate buffer (pH 6.8) at a flow rate of 0.8 mL/min. Adenosine phosphate content was detected at a wavelength of 254 nm.

### 2.8. Analysis of Glycolytic Enzyme Activity

Creatine kinase (CK), phosphofructokinase muscle (PFKM), hexokinase (HK), lactate dehydrogenase (LDH), malate dehydrogenase (MDH), and pyruvate kinase (PK) activities were analyzed using a colorimetric method according to the manufacturer’s instructions accompanying the respective kits (Nanjing Jiancheng Bioengineering Institute, Nanjing, China). In brief, 1 g frozen meat samples were added to 9 mL of extraction buffer (80 mmol/L K_2_HPO_4_, 15 mmol/L KH_2_PO_4_, 25 mmol/L KCl, 1.2 mol/L NaCl, pH 7.4, 4 °C), homogenized in an ice bath, and separated via centrifugation at 13,000× *g* for 10 min at 4 °C. Thereafter, the activities of CK, HK, LDH, MDH, PFKM, and PK in the resulting supernatants were measured using a UV-2450 spectrophotometer (Shimadzu, Kyoto, Japan) according to the manufacturer’s instructions. Further, the protein contents of the supernatants were measured using a bicinchoninic acid protein quantification kit (Nanjing Jiancheng Bioengineering Institute). One unit of glycolytic enzyme activity was expressed as the quantity of enzyme that catalyzed the production of 1 μmol products min^−1^·g pro^−1^ from the substrate [22].

### 2.9. Transcriptome Analysis

#### 2.9.1. RNA Extraction, Library Construction, and RNA Sequencing

Total RNA was extracted from LTM samples of the AC2 and CN groups using TRIzol reagent (Invitrogen, Carlsbad, CA, USA). The quality and quantity of the obtained RNA were evaluated using agarose gel electrophoresis and spectrophotometry (NanoDrop 2000, Thermo Scientific, Waltham, MA, USA).

The LTM mRNA was enriched using oligo(dT)-conjugated magnetic beads in the NEBNext Ultra II RNA Library Prep Kit for Illumina (New England Biolabs, Ipswich, MA, USA). Thereafter, first-strand cDNA was synthesized using random hexamers of the obtained mRNA, and the second strand of cDNA was synthesized using DNA polymerase I. Double-stranded cDNA was purified using AMPure XP beads and subjected to end repair, the addition of poly(A) tail, and PCR amplification. Thus, the cDNA library was obtained. After quality assessment, the cDNA library was sequenced on an Illumina HiSeq 6000 platform (Illumina, San Diego, CA, USA) at Shanghai Personal Biotechnology Co., Ltd. (Shanghai, China).

#### 2.9.2. Identification of Differentially Expressed Genes (DEGs)

The reference pig (Sus scrofa) genome and transcript annotation files (Sscrofa11.1, GCA_000003025.6) were downloaded from NCBI (https://www.ncbi.nlm.nih.gov/datasets/genome/?taxon=9823, accessed on 11 December 2023). Further, clean reads were obtained by filtering the raw data using FastQC software (https://www.bioinformatics.babraham.ac.uk/projects/fastqc/, accessed on 11 December 2023), and to match the clean reads to the reference genome to acquire transcript location and sequence information, HISAT2 software (v2.1.0) was used [23]. The matched reads were then used for transcriptome assembly using StringTie software (v2.2.1) [24]. DEGs between the AC2 and CN groups were then identified using R package DESeq2 (v.1.30.1) with *P*_adj_ < 0.05 and |log2(fold change)| > 1 as selection criteria [25].

#### 2.9.3. Functional Enrichment Analysis of DEGs

Gene Ontology (GO) functional annotation and Kyoto Encyclopedia of Genes and Genomes (KEGG) pathway enrichment analyses of the DEGs were conducted and visualized using ClusterProfiler [26]. Pathways were considered significantly enriched at *P*_adj_ < 0.05.

### 2.10. Quantitative Real-Time PCR (qPCR)

Total RNA was extracted from LTM samples using TRIzol reagent (Invitrogen), following the manufacturer’s instructions. After determining the quality and quantity of the total RNA, first-strand cDNA was synthesized according to the instructions accompanying the manufacturer’s kit (PrimeScript RT reagent Kit with gDNA Eraser, TaKaRa). qPCR was then performed on a QuantStudio 6 Flex Real-Time PCR System (ABI, Carlsbad, CA, USA) using TB Green Premix Ex Taq II (TaKaRa, Beijing, China), following the manufacturer’s instructions. Relative gene expression levels were determined using the 2^−ΔΔCt^ method with glyceraldehyde-3-phosphate dehydrogenase (*GAPDH*) as the reference gene. The primer sequences used for qPCR are listed in Table S1.

### 2.11. Statistical Analysis

All the data on meat quality, amino acid contents, myofiber diameter, density, cross-sectional area, GP and adenosine phosphate content, and glycolytic enzyme activity were analyzed in SPSS software version 26.0 for Windows (IBM, Chicago, IL, USA). Further, DEG validation data were analyzed using a two-tailed Student’s *t*-test; while other data were analyzed via a one-way ANOVA with Tukey’s multiple comparison test using SPSS software version 26.0 (IBM). All data are presented as means ± SEM, and statistical significance was set at *p* < 0.05.

## 3. Results

### 3.1. Meat Quality

As shown in Table 2, there were no differences in LTM pH_45min_, b*_45min_, b*_24h_, moisture, protein, ash, and drip loss among the three groups (*p* > 0.05). In contrast, relative to the CN group, the AC2 group showed significantly higher pH_24h_, a*_45min_, a*_24h_, and IMF values (*p* < 0.05) and significantly lower L*_45min_, L*_24h_, cooking loss, and shear force values (*p* < 0.05). Further, relative to the AC1 group, the AC2 group showed significantly higher a*_24h_ and IMF values and significantly lower L*_45min_, L*_24h_, cooking loss, and shear force values (*p* < 0.05). However, no significant differences were observed between the AC1 and CN groups with respect to these indicators (*p* > 0.05).

### 3.2. Amino Acid Analysis

As shown in Table 3, Glu was the most abundant amino acid in the LTM samples from all the different groups, followed by Asp, Lys, and Leu. No significant differences were observed among the three groups with respect to the total amino acid (TAA) content, essential amino acid (EAA) content, and the EAA-to-TAA and umami amino acid (UAA)-to-TAA ratios (*p* > 0.05). In contrast, relative to the CN group, the AC2 group showed significantly higher Asp, Glu, and UAA contents (*p* < 0.05). However, no notable differences in amino acid contents were observed between the AC1 and CN groups.

### 3.3. GP, Glycolytic Enzyme Activities, and Adenosine Phosphate Levels

Table 4 shows the effects of dietary acorn supplementation on GP, glycolytic enzyme activity, and the adenosine phosphate content of the finishing Yuxi pigs. From this table, it is evident that at 24 h postmortem, CN pigs showed significantly higher glycogen, ATP, and ADP levels, and CK activity than AC2 pigs (*p* < 0.05). However, GP, lactic acid content, and LDH, MDH, PFKM, and PK activities exhibited an opposite trend (*p* < 0.05). Further, no significant differences in AMP concentration or HK activity were observed among the different groups, and except for GP and PFKM activity, no other differences were observed between the AC1 and CN groups (*p* > 0.05).

### 3.4. Myofiber Histology

**Figure 1:**
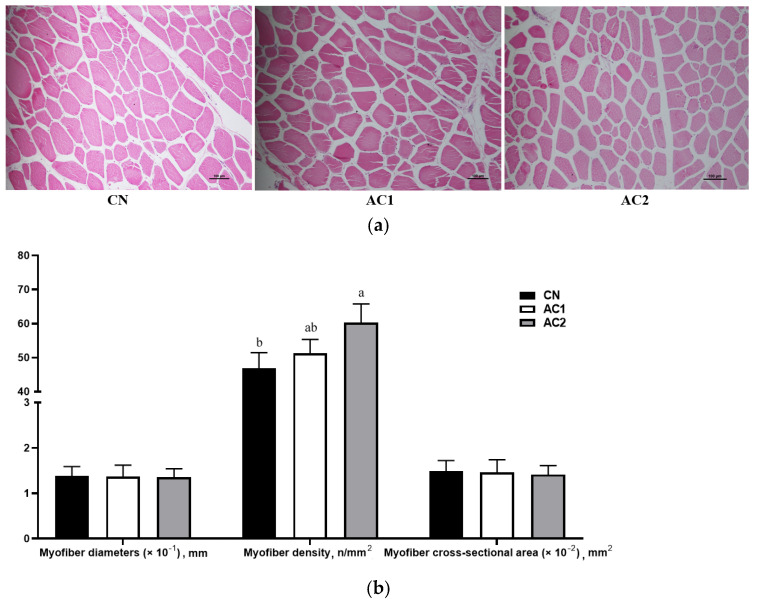
The effects of acorns on the morphology of the *Longissimus thoracis* muscle in finishing Yuxi pigs. (**a**) Section stained with hematoxylin–eosin; (**b**) myofiber diameter (×10^−1^, mm), myofiber density (n/mm^2^), and myofiber cross-sectional area (×10^−2^, mm^2^). Abbreviations: AC1, group fed a diet containing 100 g/kg of acorns; AC2, group fed a diet containing 300 g/kg of acorns; and CN, group fed a commercial diet. In the histogram, columns represent means ± SEM and different lowercase letters above the columns denote significant differences (*p* < 0.05); *n* = 10.

From Figure 1a,b, it is evident that dietary acorn supplementation had no significant effect on myofiber diameter or cross-sectional area in the LTM of finishing Yuxi pigs (*p* > 0.05). However, the AC2 treatment resulted in a significant increase in LTM myofiber density relative to the CN treatment (*p* < 0.05).

### 3.5. Expression Levels of Myofiber-Related Genes

**Figure 2:**
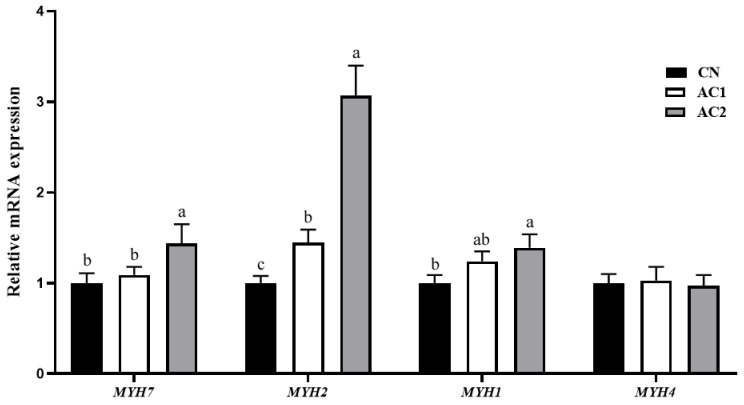
The effect of dietary acorns on the expression of myofiber-related genes in the *Longissimus thoracis* muscle of finishing Yuxi pigs. *GAPDH* served as the housekeeping gene. In the histogram, different lowercase letters above the columns denote significant differences (*p* < 0.05). Abbreviations: AC1, group fed a diet containing 100 g/kg of acorns; AC2, group fed a diet containing 300 g/kg of acorns; CN, group fed a commercial diet; *GAPDH*, glyceraldehyde-3-phosphate dehydrogenase; *MYH1*, myosin heavy chain type IIx; *MYH2*, myosin heavy chain type IIa; *MYH4*, myosin heavy chain type IIb; *MYH7*, myosin heavy chain type I; and *n* = 10.

The mRNA expression levels of *MYH1*, *MYH2*, *MYH4*, and *MYH7* in the LTM of finishing Yuxi pigs are shown in Figure 2. Compared to the CN treatment, the AC2 treatment significantly upregulated *MYH7*, *MYH2*, and *MYH1* gene expression levels in the LTM of the treated pigs (*p* < 0.05), whereas the gene expression levels of *MYH7* and *MYH2* in the LTM of pigs in the AC2 group were significantly higher than those of pigs in the AC1 group (*p* < 0.05). However, no notable differences in *MYH4* mRNA levels were observed among the three groups (*p* > 0.05). Moreover, except for *MYH2* (*p* < 0.05), no significant differences were observed between the AC1 and CN groups with respect to the mRNA levels of myofiber-related genes (*p* > 0.05).

### 3.6. Transcriptome Analysis

The data obtained from transcriptome sequencing were subjected to quality control and filtering. As shown in Tables S2 and S3, a total of 251.8 G clean reads were obtained from six samples (three each from the CN and AC2 groups), representing over 37.8 G per sample. Q30 was greater than 95.86%, thus the requirements for bioinformatics analysis were met. As shown in Table S4, the total number of clean read sequences mapped to the reference genome (Sscrofa11.1) exceeded 97.36%, indicating the reliability of our data and their applicability in further analyses.

### 3.7. DEG Screening and Analysis

**Figure 3:**
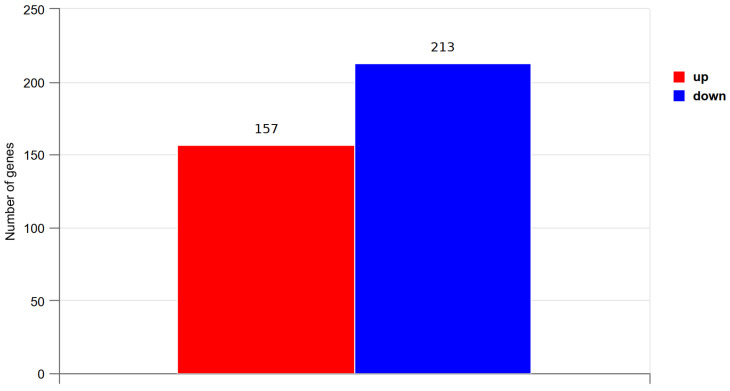
The differentially expressed genes (DGEs) in the *Longissimus thoracis* muscle between the AC2 and CN groups. Abbreviations: AC2, group fed a diet containing 300 g/kg of acorns; CN, group fed a commercial diet; and *n* = 3.

**Figure 4:**
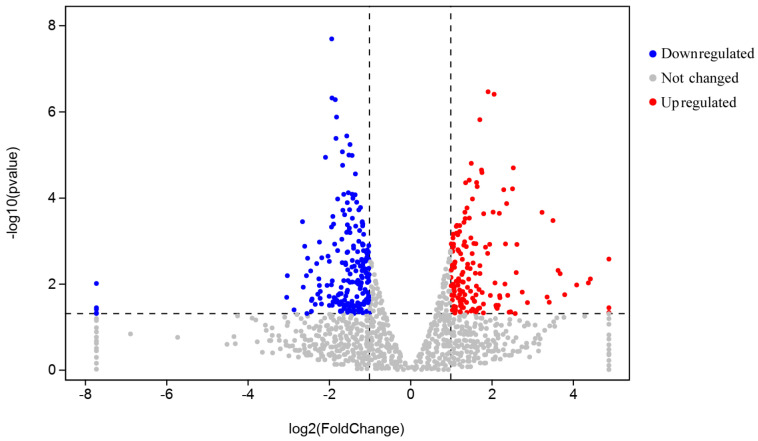
A volcano map of the differentially expressed genes (DGEs) in the *Longissimus thoracis* muscle between the AC2 and CN groups. Abbreviations: AC2, group fed a diet containing 300 g/kg of acorns; CN, group fed a commercial diet; and *n* = 3.

The DEGs between the LTM of pigs in the CN and AC2 groups were screened using DESeq2 according to the criteria: *P*_adj_ < 0.05 and |log2(fold change)| > 1. As shown in Figure 3 and Figure 4, and Table S5, 370 DEGs were identified (157 upregulated and 213 downregulated in the AC2 group relative to the CN group). Among the top 10 DEGs, 7 (thy-1 cell surface antigen [*THY1*], myristoylated alanine-rich protein kinase C substrate [*MARCKS*], collagen type III alpha 1 chain [*COL3A1*], parvalbumin [*PVALB*], CYFIP related Rac1 interactor A [*CYRIA*], angiopoietin-like 1 [*ANGPTL1*], and fibronectin 1 [*FN1*]) were downregulated, while 3 (suppressor of cytokine signaling 3 [*SOCS3*], citron rho-interacting serine/threonine kinase [*CIT*], and solute carrier family 2 member 5 [*SLC2A5*]) were upregulated.

**Figure 5:**
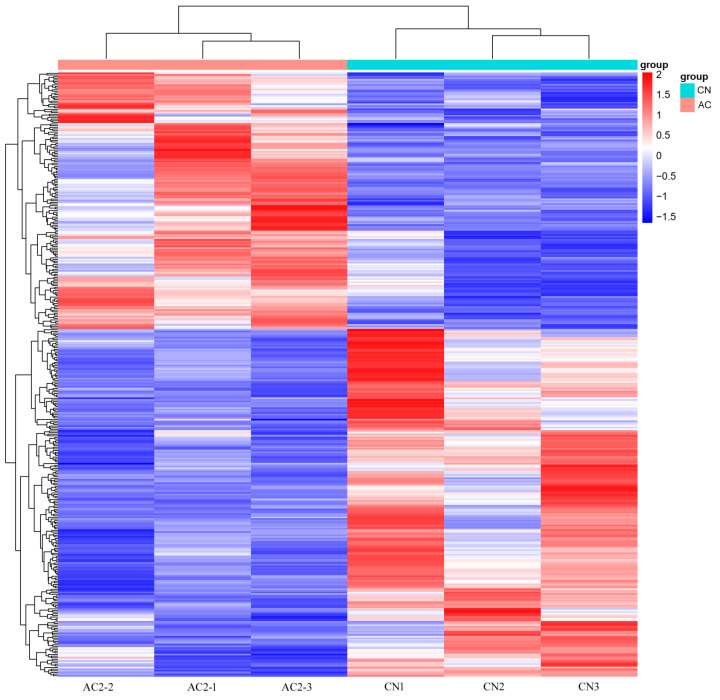
A heatmap of the stratified clustering of the differentially expressed genes (DGEs) in the *Longissimus thoracis* muscle between the AC2 and CN groups. Abbreviations: AC2, group fed a diet containing 300 g/kg of acorns; and CN, group fed a commercial diet. Red indicates upregulated DGEs and blue indicates downregulated DGEs; *n* = 3.

Additionally, the clustering analysis of the DEGs (Figure 5) showed that the two groups of samples clustered into two branches, and DEGs with similar expression patterns also clustered together, consistent with the biological grouping. Thus, the identified DEGs were suitable for further analysis.

### 3.8. GO Annotation and KEGG Pathway Mapping

**Figure 6:**
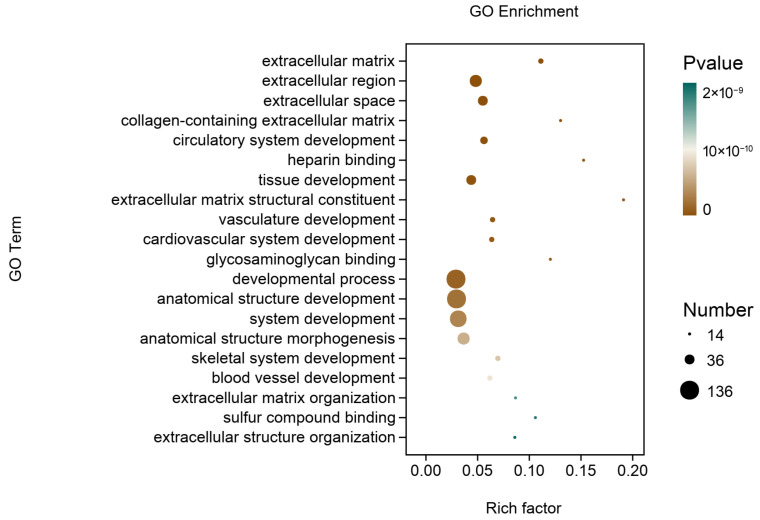
Gene Ontology (GO) term enrichment analysis of the differentially expressed genes (DEGs) of the *Longissimus thoracis* muscle (top 20 GO terms; *n* = 3).

**Figure 7:**
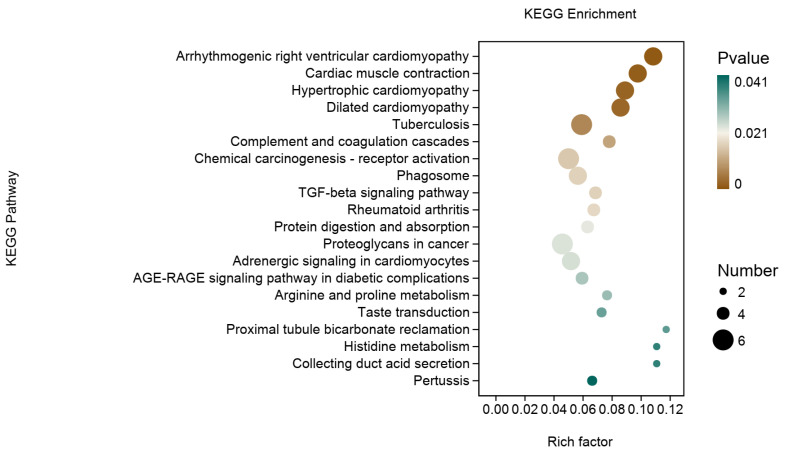
Kyoto Encyclopedia of Genes and Genomes (KEGG) pathway enrichment analysis of the differentially expressed genes (DEGs) in the *Longissimus thoracis* muscle (the top 20 enriched pathways; *n* = 3).

The functional annotation of the DEGs revealed significant enrichment in 5447 GO entries, including 4348 biological processes, 420 cellular components, and 679 molecular functions. The top 20 GO terms ranked based on -log10 values (*p*-value) were principally related to extracellular matrix and tissue development (Figure 6). Further, as shown in Figure 7, the KEGG pathway enrichment analysis indicated that the DEGs were mainly associated with human diseases, cell function, signal transduction, and substance and energy metabolism. Interestingly, the DEGs were also enriched in pathways related to the metabolism of umami amino acids, such as glutamate, aspartate, alanine, and glycine.

### 3.9. DEG Validation

**Figure 8:**
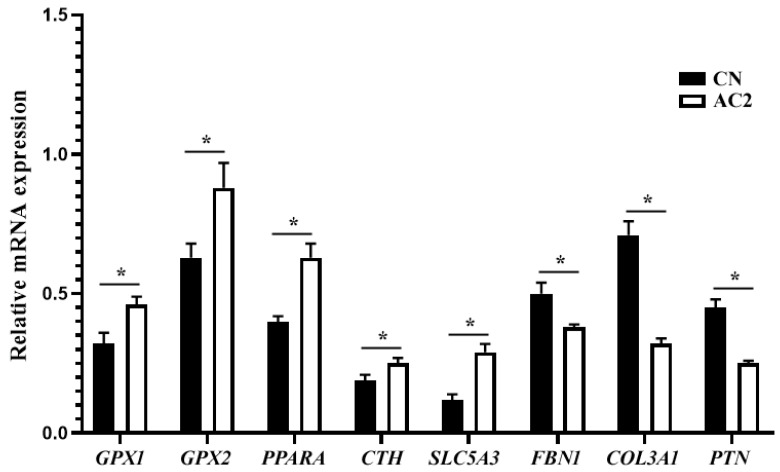
qPCR-based verification of the sequencing results for eight randomly selected differentially expressed genes (DGEs) in the *Longissimus thoracis* muscle. Abbreviations: AC2, group fed a diet containing 300 g/kg of acorns; CN, group fed a commercial diet. *GAPDH* served as the housekeeping gene. In the histogram, an asterisk (*) above the columns denotes a significant difference (*p* < 0.05). *COL3A1*, collagen type III alpha 1 chain; *CTH*, cystathionine gamma-lyase; *FBN1*, fibrillin 1; *GAPDH*, glyceraldehyde-3-phosphate dehydrogenase; *GPX1*, glutathione peroxidase 1; *GPX2*, glutathione peroxidase 2; *PPARA*, peroxisome proliferator activated receptor alpha; *PTN*, pleiotrophin; *SLC5A3*, solute carrier family 5 member 3; and *n* = 10.

Eight DEGs were randomly selected for the validation of transcriptome sequencing via qPCR. As shown in Figure 8, the mRNA expression levels of five of the eight DEGs, *GPX1*, *GPX2*, *PPARA*, *CTH*, and *SLC5A3* in the LTM of AC2 pigs were significantly upregulated, whereas those of the three other DEGs, *FBN1*, *COL3A1*, and *PTN*, were significantly downregulated relative to their levels in LTM from pigs in the CN group (*p* > 0.05). These observations are consistent with the transcriptome sequencing results, and thus validate the reliability of the transcriptome sequencing data.

## 4. Discussion

Acorns, which constitute a vital wild woody food resource globally and have a starch content of 50–70%, have been a feed resource for pigs since ancient times [3,27]. Meat quality traits are critical indicators of pork quality and directly affect economic benefits to pig farmers [28]. Further, meat color, which is the key determinant of consumer preference during purchase, is primarily determined by myoglobin and hemoglobin contents. The pH of postmortem meat is also an important indicator of the rate of muscle glycogen fermentation, and an increase in pH after slaughter is advantageous for pork tenderness and flavor [29]. IMF is also a critical trait that determines meat quality, including flavor, juiciness, and tenderness [6]. The shear force of meat is also reflective of muscle tenderness. In this study, a significant increase in a*_45min_, a*_24h_, pH_24h_, and IMF, and a decline in cooking loss and shear force were observed after feeding the pigs diet containing 300 g/kg of acorns. This observation indicated that feeding pigs a 300 g/kg acorn diet can improve pork quality. However, our findings regarding meat color were inconsistent with previously reported findings [7,30]. These differences may be due to the antioxidant properties of the tannins in acorns as well as differences in tannin contents.

Amino acids, the basic units of proteins, determine the nutritional value of pork and affect its taste and flavor, and umami amino acids in particular play a critical role in this regard [31,32]. Glu and Asp are well-known umami amino acids in meat [31]. In this study, we observed that the AC2 treatment improved the taste and flavor of pork from finishing Yuxi pigs by increasing the contents of Asp, Glu, and UAA, suggesting that an additional dose of 300 g/kg of acorns in pigs’ diets could improve pork taste and flavor.

Muscle glycolysis and related enzymes are critical for maintaining postmortem meat quality. For example, postmortem pH is controlled by the rates of glycolysis and ATP hydrolysis. It is also widely known that CK is an important indicator for evaluating the energy supply capacity of the phosphate system. Additionally, LDH, PFKM, and PK are key enzymes that control glycolysis, while MDH activity is reflective of the aerobic metabolic capacity of muscles [20,33]. Our results revealed a remarkable increase in the content of glycogen, ATP, ADP, and CK activity and a decrease in the lactic acid content and activities of LDH, MDH, PFKM, and PK in LTM from pigs in the AC2 group. Therefore, we speculated that 300 g/kg acorn increased LTM postmortem pH_24h_ by regulating glycolysis and the activity levels of related enzymes, GP, and adenosine phosphate, thereby improving pork tenderness and water-holding capacity. Our results are consistent with those of Chen et al. [34] and Wang et al. [35].

It has also been reported that myofiber traits, such as IMF, pH, color, and meat tenderness, which are primarily determined by myofiber diameter, density, cross-sectional area, and type, have significant effects on glycolysis and postmortem meat quality [36]. According to myosin heavy chain (MyHC) polymorphisms, myofibers, which are divided into four types, namely: type I or slow oxidative, type IIa or fast oxidoglycolytic, type IIb or fast glycolytic, and type IIx or fast oxidative, are encoded by the *MYH7*, *MYH2*, *MYH4*, and *MYH1* genes, respectively [37]. Notably, MyHC IIb has a lower myoglobin content than MyHC I and IIa; therefore, MyHC I and IIa can be converted into MyHC IIb, leading to a lighter color, a decline in pH, and higher drip loss [29]. Previous studies have also demonstrated that MyHC I and IIa are beneficial for postmortem meat color, ultimate pH, tenderness, and water-holding capacity, whereas MyHC IIb has the opposite effect on these meat quality indicators [28,38,39]. Additionally, nutrition, which is the material basis for muscle growth and development, directly affects the composition of animal myofibers [40]. Similarly, our results revealed that the dietary addition of 300 g/kg of acorns upregulated the mRNA expression levels of *MYH7*, *MYH2*, and *MYH1* and improved myofiber density in the LTM of finishing Yuxi pigs. This observation indicated that acorns might induce the transformation of myofiber types from MyHC IIb to MyHC I and MyHC IIa, and also bring about an increase in intramuscular fat content, thereby improving meat quality. However, the underlying mechanism remains unclear and, thus, requires further investigation.

In this study, we also analyzed LTM from finishing Yuxi pigs fed acorns via RNA-seq to identify the pathways and candidate genes involved in meat quality improvement under the AC2 treatment. A total of 370 DEGs (157 upregulated and 213 downregulated in the AC2 group) were identified, and the top 10 DEGs were found to be mainly involved in the cytoskeleton formation, cytomatrix formation, cell function, angiogenesis, energy metabolism, and signal transduction. The extracellular matrix, a non-cellular three-dimensional network, primarily consists of collagen and plays a vital role in muscle nutrition and myocyte dynamics regulation. Thus, it affects meat quality parameters, such as tenderness and water-holding capacity [41,42]. The *COL3A1* gene encodes type III collagen, a major component of the extracellular matrix of connective tissue in muscles, skin, and blood vessels, among other organs [43]. Bao et al. [42] reported that *COL3A1* expression is negatively associated with collagen solubility, which contributes to meat tenderness. *SOCSs* are important negative regulators of cytokine signaling and are associated with the blocking of leptin and insulin signaling, both of which play key roles in the regulation of energy metabolism [44]. It has also been shown that *CIT* regulates myosin contractility and participates in the regulation of cytokinesis by phosphorylating myosin regulatory light chains [45]. *PVALB* is a high-affinity calcium-binding protein involved in muscle tissue relaxation processes [46]. One study showed that the *PVALB* expression level is negatively associated with meat quality [47]. In this study, we observed that dietary supplementation with 300 g/kg of acorns significantly downregulated the expression levels of *COL3A1* and *PVALB* and upregulated those of *SOCS3* and *CIT*, ultimately affecting meat quality by regulating extracellular matrix production and energy metabolism. These findings highlight the complexity of the processes underlying meat quality. Furthermore, we speculated that the pigs in the AC groups may suppress the secretion of leptin by upregulating the expression of *SOCS3*, leading to more IMF in the *Longissimus thoracis* muscles. The GO enrichment analysis indicated that the top 20 DEGs were mainly related to extracellular matrix and tissue development, whereas the KEGG pathway analysis showed that the DEGs were mainly associated with pathways related to human diseases, cell function, signal transduction, and substance and energy metabolism, particularly the upregulation of glutamate, aspartate, and glycine metabolism. These findings suggested that dietary supplementation with 300 g/kg of acorns increased Asp, Glu, and umami amino acid contents, thereby improving the taste and flavor of pork from finishing Yuxi pigs. The underlying mechanisms may be the above-mentioned pathways and candidate genes. However, the reason for the differential expression of genes associated with KEGG pathways related to human diseases requires further investigation.

## 5. Conclusions

In this study, we investigated the effects of dietary acorn supplementation on pork quality in finishing Yuxi pigs. Our results indicated that dietary supplementation with 300 g/kg of acorns in finishing Yuxi pigs improved pork quality by inducing myofiber conversion to types that improve pork quality and taste, regulating glycolysis, extracellular matrix formation, and substance and energy metabolism. The findings of this study highlight acorn supplementation as a strategy for improving pork quality and a basis for the development of alternative feed components enhancing the sustainability of the swine industry.

## Figures and Tables

**Table 1:** Composition and nutrition levels of the diets.

Item	CN	AC1	AC2
Ingredient, %			
Corn	61.70	52.50	37.10
Soybean meal	10.70	13.10	17.20
Wheat bran	20.65	16.42	6.00
Acorn	0.00	10.00	30.00
Soybean oil	2.92	3.95	5.67
CaHPO_4_	1.30	1.30	1.30
Limestone	1.15	1.15	1.15
Salt	0.30	0.30	0.30
Lysine	0.18	0.18	0.18
Methionine	0.10	0.10	0.10
Premix ^1^	1.00	1.00	1.00
Total	100.00	100.00	100.00
Nutrient level ^2^			
Digestible energy, MJ·kg^−1^	13.42	13.42	13.41
Crude protein, %	12.93	12.94	12.94
Calcium, %	0.95	0.95	0.94
Total phosphorus, %	0.69	0.68	0.67
Available phosphorous, %	0.38	0.37	0.36
Methionine, %	0.30	0.29	0.29
Lysine, %	0.84	0.85	0.85
SID lysine, %	0.76	0.76	0.76

^1^ The premix provided the following per kg of diet: vitamin A, 3000 IU; vitamin B_1_, 3 mg; vitamin B_2_, 5 mg; vitamin B_6_, 3.2 mg; vitamin B_12_, 0.03 mg; vitamin D_3_, 300 IU; vitamin E, 22.5 mg; vitamin K_3_, 2 mg; folic acid, 1.5 mg; nicotinic acid, 25 mg; pantothenic acid, 15 mg; Cu, 5 mg; Fe, 80 mg; I, 0.4 mg; Mn, 20.5 mg; Se, 0.25 mg; and Zn, 80 mg. ^2^ ME was a calculated value and others were measured values. Abbreviations: AC1, group fed a diet containing 100 g/kg of acorns; AC2, group fed a diet containing 300 g/kg of acorns; CN, group fed a commercial diet; and SID lysine, standardized ileal digestible lysine.

**Table 2:** The effects of acorn on the meat quality parameters of the *Longissimus thoracis* muscle in finishing Yuxi pigs.

Item	CN	AC1	AC2	SEM	*p*-Value
pH_45min_	5.98	5.96	6.03	0.14	0.260
pH_24h_	5.64 ^b^	5.73 ^ab^	5.89 ^a^	0.09	0.023
L*_45min_	35.2 ^a^	35.1 ^a^	31.5 ^b^	1.26	0.012
a*_45min_	7.0 ^b^	7.3 ^b^	9.7 ^a^	0.78	0.005
b*_45min_	3.5	3.5	3.2	0.56	0.466
L*_24h_	40.8 ^a^	40.0 ^a^	34.9 ^b^	1.73	0.001
a*_24h_	7.9 ^b^	8.2 ^b^	10.3 ^a^	0.17	<0.001
b*_24h_	4.8	4.7	4.5	0.52	0.314
Moisture, %	68.7	67.8	67.5	1.82	0.233
Protein, %	22.1	22.5	22.0	0.56	0.108
IMF, %	4.3 ^c^	5.5 ^b^	7.4 ^a^	0.45	<0.001
Ash, %	1.1	1.1	1.0	0.13	0.204
Drip loss, %	3.3	3.4	3.1	0.27	0.174
Cooking loss, %	27.7 ^a^	27.5 ^a^	25.7 ^b^	1.31	0.016
Shear force, N	56.9 ^a^	55.0 ^a^	38.8 ^b^	2.58	<0.001

In the same row, values with different lowercase letters denote significance from each other (*p* < 0.05). Abbreviations: AC1, group fed a diet containing 100 g/kg of acorns; AC2, group fed a diet containing 300 g/kg of acorns; CN, group fed a commercial diet; L*, lightness; a*, redness; b*, yellowness; 45 min and 24 h, 45 min, and 24 h postmortem, respectively; IMF, intramuscular fat; and *n* = 10.

**Table 3:** The effects of acorn on the amino acid composition of the *Longissimus thoracis* muscle in finishing Yuxi pigs (g/100 g dry matter).

Item	CN	AC1	AC2	SEM	*p*-Value
Asp	7.33 ^b^	7.33 ^b^	7.56 ^a^	0.13	0.024
Glu	12.32 ^b^	12.48 ^ab^	12.73 ^a^	0.24	0.035
Gly	3.34	3.36	3.37	0.14	0.861
Tyr	3.47	3.50	3.53	0.13	0.170
Ala	4.37	4.33	4.34	0.08	0.166
Phe	3.81	3.82	3.84	0.07	0.223
Thr	3.64	3.61	3.58	0.23	0.240
Val	4.19	4.22	4.25	0.08	0.150
Met	2.18	2.13	2.17	0.07	0.202
Ile	4.19	4.18	4.22	0.29	0.207
Leu	6.73	6.78	6.79	0.43	0.117
Lys	7.26	7.29	7.32	0.44	0.107
Trp	1.82	1.83	1.85	0.13	0.145
His	4.62	4.54	4.40	0.08	0.118
Arg	4.87	4.84	4.88	0.08	0.445
Pro	2.80	2.81	2.72	0.10	0.350
Ser	2.80	2.74	2.57	0.14	0.146
Cys	0.78	0.78	0.76	0.03	0.611
TAA	80.52	80.57	80.88	2.87	0.324
EAA	33.82	33.86	34.02	0.79	0.113
UAA	34.64 ^b^	34.82 ^ab^	35.37 ^a^	0.51	0.026
EAA/TAA, %	42.00	42.03	42.06	1.55	0.132
UAA/TAA, %	43.02	43.22	43.73	1.24	0.221

In the same row, values with different lowercase letters denote significance from each other (*p* < 0.05). Abbreviations: AC1, group fed a diet containing 100 g/kg of acorns; AC2, group fed a diet containing 300 g/kg of acorns; CN, group fed a commercial diet; EAA, essential amino acids (Leu, Ile, Lys, Met, Thr, Trp, Phe, and Val); TAA, total amino acids; UAA, umami amino acids (Ala, Asp, Glu, Gly, Phe, and Tyr); and *n* = 10.

**Table 4:** The effects of acorns on the glycolytic potential, enzyme activities, and adenosine phosphate contents in the *Longissimus thoracis* muscle of finishing Yuxi pigs.

Items	CN	AC1	AC2	SEM	*p*-Value
Glycogen, µmol/g	9.85 ^b^	10.32 ^b^	14.16 ^a^	1.27	0.031
Lactic acid, µmol/g	75.03 ^a^	72.08 ^a^	51.92 ^b^	4.68	<0.001
GP, µmol/g	94.71 ^a^	89.42 ^b^	80.25 ^b^	5.07	0.014
CK, U/g	4.21 ^b^	4.84 ^b^	6.77 ^a^	0.89	0.015
HK, U/g	47.06	47.19	45.11	3.52	0.256
LDH, U/g	15.63 ^a^	14.46 ^a^	10.37 ^b^	1.44	0.020
MDH, U/g	27.09 ^a^	26.01 ^a^	22.89 ^b^	2.61	0.003
PFKM, U/g	7.15 ^a^	6.13 ^b^	3.56 ^c^	1.15	0.012
PK, U/g	46.80 ^a^	44.56 ^a^	33.24 ^b^	4.33	0.001
ATP, µmol/g	0.14 ^b^	0.15 ^b^	0.19 ^a^	0.04	0.025
ADP, µmol/g	0.23 ^b^	0.21 ^b^	0.32 ^a^	0.05	0.003
AMP, µmol/g	0.09	0.11	0.11	0.02	0.156

In the same row, values with different lowercase letters denote significance from each other (*p* < 0.05). Abbreviations: AC1, group fed a diet containing 100 g/kg of acorns; AC2, group fed a diet containing 300 g/kg of acorns; CN, group fed a commercial diet; ATP, adenosine triphosphate; ADP, adenosine diphosphate; AMP, adenosine monophosphate; CK, creatine kinase; GP, glycolytic potential; HK, hexokinase; LDH, lactate dehydrogenase; MDH, malate dehydrogenase; PFKM, phosphofructokinase, muscle; PK, pyruvate kinase; and *n* = 10.

## Data Availability

The datasets that support the findings of this study are available from the corresponding author upon reasonable request.

## Supplementary Materials

The following supporting information can be downloaded at https://www.mdpi.com/article/10.3390/ani15050614/s1: Table S1. The primers used for qPCR. Table S2. Statistical results of transcriptome sequencing raw data in *Longissimus thoracis* muscle of finishing Yuxi pigs. Table S3. Statistical results of quality control of transcriptome sequencing data in *Longissimus thoracis* muscle of finishing Yuxi pigs. Table S4. Statistical results of transcriptome sequence alignment in *Longissimus thoracis* muscle of finishing Yuxi pigs. Table S5. The differentially expressed genes.

## References

[1] Makkar H.P.S. (2018). Review: Feed demand landscape and implications of food-not feed strategy for food security and climate change. Animal.

[2] Lebret B., Lhuisset S., Labussière E., Louveau I. (2023). Combining pig genetic and feeding strategies improves the sensory, nutritional and technological quality of pork in the context of relocation of feed resources. Meat Sci..

[3] Wang Y., Xu C., Wang Q., Jiang Y., Qin L. (2022). Germplasm resources of oaks (*Quercus* L.) in China: Utilization and prospects. Biology.

[4] Çetin N., Ciftci B., Kara K., Kaplan M. (2023). Effects of gradually increasing drying temperatures on energy aspects, fatty acids, chemical composition, and in vitro ruminal fermentation of acorn. Environ. Sci. Pollut. Res. Int..

[5] Li S., Zhou Y., Liu M., Zhang Y., Cao S. (2015). Nutrient composition and starch characteristics of *Quercus glandulifera* Bl. seeds from China. Food Chem..

[6] Rodríguez-Sánchez J.A., Ripoll G., Latorre M.A. (2010). The influence of age at the beginning of Montanera period on meat characteristics and fat quality of outdoor Iberian pigs. Animal.

[7] Tejeda J.F., Hernández-Matamoros A., Paniagua M., González E. (2020). Effect of free-range and low-protein concentrated diets on growth performance, carcass traits, and meat composition of Iberian pig. Animals.

[8] Fernández-Fígares I., Rodríguez-López J.M., González-Valero L., Lachica M. (2018). Iberian pig adaptation to acorn consumption: I. Net portal appearance of metabolites. PeerJ.

[9] Lachica M., Rodríguez-López J.M., González-Valero L., Fernández-Fígares I. (2018). Iberian pig adaptation to acorn consumption: II. Net portal appearance of amino acids. PeerJ.

[10] Tejerina D., García-Torres S., Cabeza de Vaca M., Vázquez F.M., Cava R. (2012). Effect of production system on physical-chemical, antioxidant and fatty acids composition of Longissimus dorsi and Serratus ventralis muscles from Iberian pig. Food Chem..

[11] Garrido N., Izquierdo M., Hernández-García F.I., Núñez Y., García-Torres S., Benítez R., Padilla J.Á., Óvilo C. (2023). Differences in muscle lipogenic gene expression, carcass traits and fat deposition among three Iberian pig strains finished in two different feeding systems. Animals.

[12] Sun Z., Liu D., An S., Wu X., Zhang J., Miao Z. (2024). Effects of acorns on fatty acid composition and lipid metabolism in adipose tissue of Yuxi black pigs. Animals.

[13] Qiao R., Li X., Han X., Wang K., Lv G., Ren G., Li X. (2019). Population structure and genetic diversity of four Henan pig populations. Anim. Genet..

[14] (2020). Nutrient Requirements of Swine.

[15] Ortiz A., Tejerina D., García-Torres S., González E., Morcillo J.F., Mayoral A.I. (2021). Effect of animal age at slaughter on the muscle fibres of *Longissimus thoracis* and meat quality of fresh loin from Iberian × Duroc crossbred pig under two production systems. Animals.

[16] Association of Official Analytical Chemists (AOAC) (2000). Animal Feed.

[17] Wang Y., Ning C., Wang C., Guo J., Wang J., Wu Y. (2019). Genome-wide association study for intramuscular fat content in Chinese Lulai black pigs. Asian-Australas J. Anim. Sci..

[18] Li F., Duan Y., Li Y., Tang Y., Geng M., Oladele O.A., Kim S.W., Yin Y. (2015). Effects of dietary n-6:n-3 PUFA ratio on fatty acid composition, free amino acid profile and gene expression of transporters in finishing pigs. Br. J. Nutr..

[19] Zhang L., Yue H.Y., Zhang H.J., Xu L., Wu S.G., Yan H.J., Gong Y.S., Qi G.H. (2009). Transport stress in broilers: I. Blood metabolism, glycolytic potential, and meat quality. Poult. Sci..

[20] Li Y.J., Gao T., Li J.L., Zhang L., Gao F., Zhou G.H. (2017). Effects of dietary starch types on early postmortem muscle energy metabolism in finishing pigs. Meat Sci..

[21] Wang X., Li J., Cong J., Chen X., Zhu X., Zhang L., Gao F., Zhou G. (2017). Preslaughter transport effect on broiler meat quality and post-mortem glycolysis metabolism of muscles with different fiber types. J. Agric. Food. Chem..

[22] Chen L., Bai Y., Everaert N., Li X., Tian G., Hou C., Zhang D. (2019). Effects of protein phosphorylation on glycolysis through the regulation of enzyme activity in ovine muscle. Food Chem..

[23] Kim D., Langmead B., Salzberg S.L. (2015). HISAT: A fast spliced aligner with low memory requirements. Nat. Methods.

[24] Pertea M., Kim D., Pertea G.M., Leek J.T., Salzberg S.L. (2016). Transcript-level expression analysis of RNA-seq experiments with HISAT, StringTie and Ballgown. Nat. Protoc..

[25] Love M.I., Huber W., Anders S. (2014). Moderated estimation of fold change and dispersion for RNA-seq data with DESeq2. Genome Biol..

[26] Yu G., Wang L.G., Han Y., He Q.Y. (2012). clusterProfiler: An R package for comparing biological themes among gene clusters. Omics.

[27] Lopez-Bote C.J. (1998). Sustained utilization of the Iberian pig breed. Meat Sci..

[28] Li Y., Feng Y., Chen X., He J., Luo Y., Yu B., Chen D., Huang Z. (2024). Dietary short-term supplementation of grape seed proanthocyanidin extract improves pork quality and promotes skeletal muscle fiber type conversion in finishing pigs. Meat Sci..

[29] Huff-Lonergan E., Baas T.J., Malek M., Dekkers J.C., Prusa K., Rothschild M.F. (2002). Correlations among selected pork quality traits. J. Anim. Sci..

[30] Szyndler-Nędza M., Świątkiewicz M., Migdał Ł., Migdał W. (2021). The quality and health-promoting value of meat from pigs of the native breed as the effect of extensive feeding with acorns. Animals.

[31] Aaslyng M.D., Meinert L. (2017). Meat flavour in pork and beef—From animal to meal. Meat Sci..

[32] Xia J.Q., Liu D.Y., Liu J., Jiang X.P., Wang L., Yang S., Liu D. (2023). Sex effects on carcass characteristics, meat quality traits and meat amino acid and fatty acid compositions in a novel Duroc line pig. J. Anim. Physiol. Anim. Nutr..

[33] Li Y., He Y., Ran J., Huang Y., Li X., Jiang H., Li X., Pan Y., Zhao S., Song C. (2023). Comparison of meat quality and glycolysis potential of two hybrid pigs in three-way hybrid model. Front. Vet. Sci..

[34] Chen R., Wen C., Gu Y., Wang C., Chen Y., Zhuang S., Zhou Y. (2020). Dietary betaine supplementation improves meat quality of transported broilers through altering muscle anaerobic glycolysis and antioxidant capacity. J. Sci. Food Agric..

[35] Wang T., Li J., Shao Y., Yao W., Xia J., He Q., Huang F. (2020). The effect of dietary garcinol supplementation on oxidative stability, muscle postmortem glycolysis and meat quality in pigs. Meat Sci..

[36] Ryu Y.C., Lee M.H., Lee S.K., Kim B.C. (2006). Effects of muscle mass and fiber type composition of longissimus dorsi muscle on postmortem metabolic rate and meat quality in pigs. J. Muscle Foods.

[37] Song S., Ahn C.H., Song M., Kim G.D. (2020). Pork loin chop quality and muscle fiber characteristics as affected by the direction of cut. Foods.

[38] Ryu Y.C., Kim B.C. (2005). The relationship between muscle fiber characteristics, postmortem metabolic rate, and meat quality of pig longissimus dorsi muscle. Meat Sci..

[39] An W., Huang Z., Mao Z., Qiao T., Jia G., Zhao H., Liu G., Chen X. (2023). Dietary taurine supplementation improves the meat quality, muscle fiber type, and mitochondrial function of finishing pigs. J. Agric. Food Chem..

[40] Schiaffino S., Reggiani C. (2011). Fiber types in mammalian skeletal muscles. Physiol. Rev..

[41] McCormick R.J. (1999). Extracellular modifications to muscle collagen: Implications for meat quality. Poult. Sci..

[42] Bao X., Zeng Y., Wei S., Wang G., Liu C., Sun Y., Chen Q., Li H. (2007). Developmental changes of Col3a1 mRNA expression in muscle and their association with intramuscular collagen in pigs. J. Genet. Genomics.

[43] Chen Y., Sun Y., Li Z., Li C., Xiao L., Dai J., Li S., Liu H., Hu D., Wu D. (2021). Identification of COL3A1 variants associated with sporadic thoracic aortic dissection: A case-control study. Front. Med..

[44] Pedroso J.A.B., Ramos-Lobo A.M., Donato J. (2019). SOCS3 as a future target to treat metabolic disorders. Hormones.

[45] Sahin I., Kawano Y., Sklavenitis-Pistofidis R., Moschetta M., Mishima Y., Manier S., Sacco A., Carrasco R., Fonseca R., Roccaro A.M. (2019). Citron Rho-interacting kinase silencing causes cytokinesis failure and reduces tumor growth in multiple myeloma. Blood Adv..

[46] Wang W., Metzger J.M. (2008). Parvalbumin isoforms for enhancing cardiac diastolic function. Cell Biochem. Biophys..

[47] Piórkowska K., Żukowski K., Ropka-Molik K., Tyra M., Gurgul A. (2018). A comprehensive transcriptome analysis of skeletal muscles in two Polish pig breeds differing in fat and meat quality traits. Genet. Mol. Biol..

