# Supplementary material for: Evaluation of the Effects of Acorns on the Meat Quality and Transcriptome Profile of Finishing Yuxi Pigs
Source: Animals (Basel). 2025 Feb 20;15(5):614. doi: 10.3390/ani15050614 (PMC11898127; doi:10.3390/ani15050614)
Supplement: Supplementary file 1 [file animals-15-00614-s001.zip › Table S1-edited.pdf]

Table S1. The primers used for qPCR

Genes	ID	Primer sequence (5′–3′)	Product length (bp)
MYH7	NM_213855	GCTGAGACGGAGAATGGCAAGAC<br>GGCGTTGTCAGAGATGGAGAAGA	303
MYH2	NM_214136	AACACCAGCCTCATCAACACCAAG<br>CTCTCTACCTCACCCTCCAGTTCAC	326
MYH1	NM_001104951	GGCTGGCTGGACAAGAACAAGG<br>TGGGAATGAGGCATCTGACAAAG	384
MYH4	NM_001123141	CACGCTGGATGCTGAGATTAGGAG<br>TTGGTGTTGATGAGGCTGGTGTTC	256
GPX1	NM_214201	ACGCTCGGTGTATGCCTTCTC<br>CGCCATTCACCTCACACTTCTC	320
GPX2	NM_001115136	CTCGCTCTGAGGCACAACCAC<br>CGCACAGGGCTCCAGATGATG	327
PPARA	NM_001044526	TCCTGCTGGTGCTGACGAGTC<br>CCGAGAGGCACTTGTGGAAACG	233
CTH	NM_001044585	CTACAGGTCCGCATGGAGAAGC<br>TCATGATTGCCGGAAGCTCAGC	280
SLC5A3	XM_005657149	CGGTTGCAGCCCTGTTCCTTC<br>TTCGTGGGAGGTGGCGTGAG	262
COL3A 1	NM_001243297	CTCCTGGTGAGCGAGGACGGC<br>ACCGGCATGTCCCTGAGGTCC	224
FBN1	NM_001001771	CGATGTGCATGCACTTACGGC<br>GGAATGAAGCCTCGGCGACAC	218
PTN	NM_214336	AGTTGTGGATACCGCTGAAGC<br>GTTGCACGGGATCTTACATCT	196
GAPDH	NM_008084	CAAGGCTGTGGGCAAGGTCATC<br>AAGTGGTCGTTGAGGGCAATGC	279

Abbreviations: *COL3A1*, collagen type III alpha 1 chain; *CTH*, cystathionine gamma-lyase; *FBN1*, fibrillin 1; *GAPDH*, glyceraldehyde-3-phosphate dehydrogenase; *GPX1*, glutathione peroxidase 1; *GPX2*, glutathione peroxidase 2; *MYH1*, myosin heavy chain IIx; *MYH2*, myosin heavy chain IIa; *MYH4*, myosin heavy chain IIb; *MYH7*, myosin heavy chain I; *PPARA*, peroxisome proliferator-activated receptor alpha; *PTN*, pleiotrophin; and *SLC5A3*, solute carrier family 5 member 3.
