# Supplementary material for: Evaluation of the Effects of Acorns on the Meat Quality and Transcriptome Profile of Finishing Yuxi Pigs
Source: Animals (Basel). 2025 Feb 20;15(5):614. doi: 10.3390/ani15050614 (PMC11898127; doi:10.3390/ani15050614)
Supplement: Supplementary file 1 [file animals-15-00614-s001.zip › Table S2-edited.pdf]

Table S2. Statistical results of transcriptome sequencing raw data in *Longissimus thoracis* muscle of finishing Yuxi pigs

Sample	Total Bases	Total Reads	Q30	N (%)	Q30 (%)	Q20 (%)
CN1	6071501620	40208620	5848668459	0.005716	96.33	98.73
CN2	6428106844	42570244	6171723106	0.005347	96.01	98.62
CN3	6689720988	44302788	6417910801	0.005737	95.94	98.58
AC2-1	7328029094	48529994	7024703172	0.005763	95.86	98.56
AC2-2	6279580828	41586628	6026021911	0.005710	95.96	98.61
AC2-3	7679037654	50854554	7396835484	0.005800	96.33	98.74

Abbreviations: AC2, the group was fed with a diet containing 300 g/kg of acorns; CN, the group was fed a corn–soybean meal type diet; Q30, total number of bases with 99.9% or more base recognition accuracy; N (%), percentage of ambiguous bases; Q30 (%), percentage of bases with 99.9% or more base recognition accuracy; and Q20 (%), percentage of bases with 99% or more base recognition accuracy.
