# Supplementary material for: Evaluation of the Effects of Acorns on the Meat Quality and Transcriptome Profile of Finishing Yuxi Pigs
Source: Animals (Basel). 2025 Feb 20;15(5):614. doi: 10.3390/ani15050614 (PMC11898127; doi:10.3390/ani15050614)
Supplement: Supplementary file 1 [file animals-15-00614-s001.zip › Table S3-edited.pdf]

Table S3. Statistical results of quality control of transcriptome sequencing data in *Longissimus thoracis* muscle of finishing Yuxi pigs

Sample	Clean reads	Clean data	Clean reads (%)	Clean data (%)
CN1	39582662	5967629590	98.44	98.29
CN2	41829536	6306743501	98.26	98.11
CN3	43521198	6560625979	98.24	98.07
AC2-1	47668390	7187025033	98.22	98.08
AC2-2	40910768	6166999922	98.37	98.21
AC2-3	50066448	7549018990	98.45	98.31

Abbreviations: AC2, the group was fed with a diet containing 300 g/kg of acorns; CN, the group was fed a corn–soybean meal type diet; clean reads (%), clean reads/total reads; and clean data (%), clean data/total bases.
