# Supplementary material for: Evaluation of the Effects of Acorns on the Meat Quality and Transcriptome Profile of Finishing Yuxi Pigs
Source: Animals (Basel). 2025 Feb 20;15(5):614. doi: 10.3390/ani15050614 (PMC11898127; doi:10.3390/ani15050614)
Supplement: Supplementary file 1 [file animals-15-00614-s001.zip › Table S4-edited.pdf]

Table S4. Statistical results of transcriptome sequence alignment in *Longissimus thoracis* muscle of finishing Yuxi pigs

Sample	Clean Reads	Total Mapped (Total Mapped/Clean Reads)	Multiple Mapped (Multiple Mapped/Total Mapped)	Uniquely Mapped (Uniquely Mapped/Total Mapped)
CN1	39582662	38536969 (97.36%)	1438720 (3.73%)	37098249 (96.27%)
CN2	41829536	40988095 (97.99%)	1865486 (4.55%)	39122609 (95.45%)
CN3	43521198	42482880 (97.61%)	1673329 (3.94%)	40809551 (96.06%)
AC2-1	47668390	46575237 (97.71%)	1905289 (4.09%)	44669948 (95.91%)
AC2-2	40910768	40086870 (97.99%)	1744382 (4.35%)	38342488 (95.65%)
AC2-3	50066448	48864339 (97.60%)	1939408 (3.97%)	46924931 (96.03%)

Abbreviations: AC2, the group was fed with a diet containing 300 g/kg of acorns; CN, the group was fed a corn–soybean meal type diet; multiple mapped, total number of sequences mapped to multiple positions in the reference genome; total mapped, total number of sequences mapped to the reference genome; and uniquely mapped, total number of sequences mapped to a single position in the reference genome.
