# Supplementary material for: Evaluation of the Effects of Acorns on the Meat Quality and Transcriptome Profile of Finishing Yuxi Pigs
Source: Animals (Basel). 2025 Feb 20;15(5):614. doi: 10.3390/ani15050614 (PMC11898127; doi:10.3390/ani15050614)
Supplement: Supplementary file 1 [file animals-15-00614-s001.zip › Table S5-edited.pdf]

Table S5. The differentially expressed genes

	id	Regulation	Name	Description
1	ENSSSCG00000036956	Up	SOCS3	suppressor of cytokine signaling 3 [Source:VGNC Symbol;Acc:VGNC:99052]
2	ENSSSCG00000009839	Up	CIT	citron rho-interacting serine/threonine kinase [Source:VGNC Symbol;Acc:VGNC:86710]
3	ENSSSCG00000039651	Up	SLC2A5	solute carrier family 2 member 5 [Source:VGNC Symbol;Acc:VGNC:93050]
4	ENSSSCG00000033816	Up	-	zinc finger protein 177-like [Source:NCBI gene (formerly Entrezgene);Acc:100738906]
5	ENSSSCG00000015584	Up	PROX1	prospero homeobox 1 [Source:VGNC Symbol;Acc:VGNC:91837]
6	ENSSSCG00000016318	Up	ASB18	ankyrin repeat and SOCS box containing 18 [Source:VGNC Symbol;Acc:VGNC:95922]
7	ENSSSCG00000011714	Up	MED12L	mediator complex subunit 12L [Source:VGNC Symbol;Acc:VGNC:90105]
8	ENSSSCG00000036227	Up	-	-
9	ENSSSCG00000032053	Up	ST8SIA5	ST8 alpha-N-acetyl-neuraminide alpha-2,8- sialyltransferase 5 [Source:VGNC Symbol;Acc:VGNC:93520]
10	ENSSSCG00000000006	Up	PPARA	peroxisome proliferator-activated receptor alpha [Source:VGNC Symbol;Acc:VGNC:91682]
11	ENSSSCG00000010853	Up	EPHX1	epoxide hydrolase 1 [Source:NCBI gene (formerly Entrezgene);Acc:397639]
12	ENSSSCG00000050998	Up	-	-
13	ENSSSCG00000034044	Up	RASD1	ras-related dexamethasone-induced 1 [Source:VGNC Symbol;Acc:VGNC:92107]
14	ENSSSCG00000044553	Up	DDIT3	DNA damage-inducible transcript 3 [Source:NCBI gene (formerly Entrezgene);Acc:100240743]
15	ENSSSCG00000022492	Up	AMPD3	adenosine monophosphate deaminase 3 [Source:VGNC Symbol;Acc:VGNC:85288]
16	ENSSSCG00000026945	Up	LRRC2	leucine-rich repeat-containing 2 [Source:VGNC Symbol;Acc:VGNC:98079]
17	ENSSSCG00000015035	Up	C11orf52	chromosome 9 C11orf52 homolog [Source:VGNC Symbol;Acc:VGNC:86085]
18	ENSSSCG00000007554	Up	ZFAND2A	zinc finger AN1-type-containing 2A [Source:VGNC Symbol;Acc:VGNC:95134]
19	ENSSSCG00000037347	Up	-	R3H and coiled-coil domain-containing protein 1- like [Source:NCBI gene (formerly Entrezgene);Acc:110258721]
20	ENSSSCG00000005278	Up	-	-
21	ENSSSCG00000001835	Up	ABHD2	abhydrolase domain-containing 2, acylglycerol lipase [Source:VGNC Symbol;Acc:VGNC:84980]
22	ENSSSCG00000003326	Up	PEG3	paternally expressed 3 [Source:VGNC Symbol;Acc:VGNC:98530]
23	ENSSSCG00000014148	Up	TMEM161B	transmembrane protein 161B [Source:VGNC Symbol;Acc:VGNC:94102]
24	ENSSSCG00000049444	Up	-	-

25	ENSSSCG00000013374	Up	KCNC1	potassium voltage-gated channel subfamily C member 1 [Source:VGNC Symbol;Acc:VGNC:89332]
26	ENSSSCG00000003379	Up	KLHL21	kelch-like family member 21 [Source:VGNC Symbol;Acc:VGNC:89520]
27	ENSSSCG00000031614	Up	SMCO1	single-pass membrane protein with coiled-coil domains 1 [Source:VGNC Symbol;Acc:VGNC:93243]
28	ENSSSCG00000002374	Up	DLST	dihydrolipoamide S-succinyltransferase [Source:VGNC Symbol;Acc:VGNC:87338]
29	ENSSSCG00000000559	Up	RASSF8	ras association domain family member 8 [Source:VGNC Symbol;Acc:VGNC:92128]
30	ENSSSCG00000001347	Up	PPP1R10	protein phosphatase 1 regulatory subunit 10 [Source:VGNC Symbol;Acc:VGNC:91717]
31	ENSSSCG00000006391	Up	ATP1A2	ATPase Na+/K+ transporting subunit alpha 2 [Source:NCBI gene (formerly Entrezgene);Acc:396828]
32	ENSSSCG00000005449	Up	PTPN3	protein tyrosine phosphatase non-receptor type 3 [Source:VGNC Symbol;Acc:VGNC:91980]
33	ENSSSCG00000032498	Up	IDH3A	isocitrate dehydrogenase (NAD(+)) 3 catalytic subunit alpha [Source:NCBI gene (formerly Entrezgene);Acc:100157242]
34	ENSSSCG00000026754	Up	ATCAY	ATCAY kinesin light chain-interacting caytaxin [Source:VGNC Symbol;Acc:VGNC:85603]
35	ENSSSCG00000033879	Up	ZNF280B	zinc finger protein 280B [Source:HGNC Symbol;Acc:HGNC:23022]
36	ENSSSCG00000013735	Up	JUNB	JunB proto-oncogene, AP-1 transcription factor subunit [Source:VGNC Symbol;Acc:VGNC:89295]
37	ENSSSCG00000006810	Up	KCNC4	potassium voltage-gated channel subfamily C member 4 [Source:VGNC Symbol;Acc:VGNC:89335]
38	ENSSSCG00000026978	Up	ROS1	ROS proto-oncogene 1, receptor tyrosine kinase [Source:VGNC Symbol;Acc:VGNC:92411]
39	ENSSSCG00000000866	Up	MYBPC1	myosin-binding protein C1 [Source:VGNC Symbol;Acc:VGNC:90499]
40	ENSSSCG00000013432	Up	MIDN	midnolin [Source:VGNC Symbol;Acc:VGNC:90216]
41	ENSSSCG00000004218	Up	RSPO3	R-spondin 3 [Source:VGNC Symbol;Acc:VGNC:92485]
42	ENSSSCG00000014336	Up	EGR1	early growth response 1 [Source:VGNC Symbol;Acc:VGNC:87590]
43	ENSSSCG00000036136	Up	BHLHE40	basic helix–loop–helix family member e40 [Source:VGNC Symbol;Acc:VGNC:85814]
44	ENSSSCG00000003621	Up	-	zinc finger and SCAN domain-containing 20 [Source:VGNC Symbol;Acc:VGNC:95327]
45	ENSSSCG00000010798	Up	GGA2	golgi associated, gamma adaptin ear-containing, ARF-binding protein 2 [Source:VGNC Symbol;Acc:VGNC:88432]

46	ENSSSCG00000016160	Up	ERBB4	erb-b2 receptor tyrosine kinase 4 [Source:VGNC Symbol;Acc:VGNC:96284]
47	ENSSSCG00000011297	Up	ABHD5	abhydrolase domain-containing 5, lysophosphatidic acid acyltransferase [Source:VGNC Symbol;Acc:VGNC:97860]
48	ENSSSCG00000032023	Up	-	-
49	ENSSSCG00000034763	Up	IRS2	insulin receptor substrate 2 [Source:VGNC Symbol;Acc:VGNC:89214]
50	ENSSSCG00000039290	Up	MTPN	myotrophin [Source:VGNC Symbol;Acc:VGNC:90467]
51	ENSSSCG00000006718	Up	ZNF697	zinc finger protein 697 [Source:VGNC Symbol;Acc:VGNC:95294]
52	ENSSSCG00000001867	Up	PSTPIP1	proline–serine–threonine phosphatase-interacting protein 1 [Source:VGNC Symbol;Acc:VGNC:91935]
53	ENSSSCG00000017962	Up	KDM6B	lysine demethylase 6B [Source:VGNC Symbol;Acc:VGNC:89416]
54	ENSSSCG00000017283	Up	CD79B	CD79b molecule [Source:VGNC Symbol;Acc:VGNC:86430]
55	ENSSSCG00000037890	Up	ZNF853	zinc finger protein 853 [Source:HGNC Symbol;Acc:HGNC:21767]
56	ENSSSCG00000004438	Up	TSPYL1	TSPY-like 1 [Source:NCBI gene (formerly Entrezgene);Acc:100518283]
57	ENSSSCG00000050590	Up	-	-
58	ENSSSCG00000022846	Up	SLC4A3	solute carrier family 4 member 3 [Source:VGNC Symbol;Acc:VGNC:95505]
59	ENSSSCG00000009642	Up	STC1	stanniocalcin 1 [Source:VGNC Symbol;Acc:VGNC:93542]
60	ENSSSCG00000010144	Up	ACTN2	actinin alpha 2 [Source:VGNC Symbol;Acc:VGNC:85046]
61	ENSSSCG00000029268	Up	TOPAZ1	testis- and ovary-specific PAZ domain-containing 1 [Source:VGNC Symbol;Acc:VGNC:94314]
62	ENSSSCG00000014291	Up	AFF4	AF4/FMR2 family member 4 [Source:VGNC Symbol;Acc:VGNC:85169]
63	ENSSSCG00000002279	Up	GPX2	glutathione peroxidase 2 [Source:VGNC Symbol;Acc:VGNC:88653]
64	ENSSSCG00000001695	Up	VEGFA	vascular endothelial growth factor A [Source:VGNC Symbol;Acc:VGNC:94815]
65	ENSSSCG00000015850	Up	DUSP4	dual-specificity phosphatase 4 [Source:VGNC Symbol;Acc:VGNC:96248]
66	ENSSSCG00000051776	Up	-	-
67	ENSSSCG00000040433	Up	-	-
68	ENSSSCG00000004434	Up	FRK	fyn-related Src family tyrosine kinase [Source:VGNC Symbol;Acc:VGNC:88235]
69	ENSSSCG00000007288	Up	MYH7B	myosin heavy chain 7B [Source:VGNC Symbol;Acc:VGNC:95701]
70	ENSSSCG00000012915	Up	-	cardiotrophin-like cytokine factor 1 [Source:VGNC Symbol;Acc:VGNC:86725]
71	ENSSSCG00000031101	Up	METTL21C	methyltransferase-like 21C [Source:VGNC Symbol;Acc:VGNC:90159]

72	ENSSSCG00000005211	Up	CD274	CD274 molecule [Source:VGNC Symbol;Acc:VGNC:86404]
73	ENSSSCG00000017947	Up	ACADVL	acyl-CoA dehydrogenase very long chain [Source:VGNC Symbol;Acc:VGNC:97864]
74	ENSSSCG00000030395	Up	ASB5	ankyrin repeat and SOCS box-containing 5 [Source:VGNC Symbol;Acc:VGNC:95834]
75	ENSSSCG00000042355	Up	-	-
76	ENSSSCG00000021638	Up	NEU3	neuraminidase 3 [Source:VGNC Symbol;Acc:VGNC:90693]
77	ENSSSCG00000013633	Up	CARM1	coactivator-associated arginine methyltransferase 1 [Source:VGNC Symbol;Acc:VGNC:86191]
78	ENSSSCG00000033979	Up	KLHL34	kelch-like family member 34 [Source:VGNC Symbol;Acc:VGNC:89528]
79	ENSSSCG00000014396	Up	DELE1	DAP3-binding cell death enhancer 1 [Source:VGNC Symbol;Acc:VGNC:87243]
80	ENSSSCG00000029304	Up	STEAP3	STEAP3 metalloreductase [Source:VGNC Symbol;Acc:VGNC:96048]
81	ENSSSCG00000022830	Up	KANSL1L	KAT8 regulatory NSL complex subunit 1-like [Source:HGNC Symbol;Acc:HGNC:26310]
82	ENSSSCG00000040735	Up	DDAH1	dimethylarginine dimethylaminohydrolase 1 [Source:VGNC Symbol;Acc:VGNC:87200]
83	ENSSSCG00000048078	Up	-	-
84	ENSSSCG00000040569	Up	PLA2G10	group 10 secretory phospholipase A2-like [Source:NCBI gene (formerly Entrezgene);Acc:110259864]
85	ENSSSCG00000041264	Up	-	-
86	ENSSSCG00000004803	Up	ACTC1	actin alpha cardiac muscle 1 [Source:VGNC Symbol;Acc:VGNC:85040]
87	ENSSSCG00000010012	Up	SLC35E4	solute carrier family 35 member E4 [Source:VGNC Symbol;Acc:VGNC:93080]
88	ENSSSCG00000007754	Up	ITGAM	integrin subunit alpha M [Source:NCBI gene (formerly Entrezgene);Acc:397459]
89	ENSSSCG00000004789	Up	THBS1	thrombospondin 1 [Source:VGNC Symbol;Acc:VGNC:93946]
90	ENSSSCG00000015450	Up	ZNF786	zinc finger protein 786 [Source:VGNC Symbol;Acc:VGNC:95302]
91	ENSSSCG00000026248	Up	-	progestin and adipoQ receptor family member 4 [Source:NCBI gene (formerly Entrezgene);Acc:100627080]
92	ENSSSCG00000014112	Up	JMY	junction mediating and regulatory protein, p53 cofactor [Source:VGNC Symbol;Acc:VGNC:89289]
93	ENSSSCG00000017502	Up	PGAP3	post-GPI attachment to proteins phospholipase 3 [Source:VGNC Symbol;Acc:VGNC:91343]
94	ENSSSCG00000008903	Up	CRACD	capping protein inhibiting regulator of actin dynamics [Source:VGNC Symbol;Acc:VGNC:86973]
95	ENSSSCG00000029990	Up	DEFB1	defensin beta 1 [Source:VGNC Symbol;Acc:VGNC:87240]
96	ENSSSCG00000011286	Up	KLHL40	kelch-like family member 40 [Source:VGNC Symbol;Acc:VGNC:89533]

97	ENSSSCG00000017265	Up	AXIN2	axin 2 [Source:VGNC Symbol;Acc:VGNC:85708]
98	ENSSSCG00000004713	Up	TGM5	transglutaminase 5 [Source:VGNC Symbol;Acc:VGNC:93937]
99	ENSSSCG00000039823	Up	-	histone–arginine methyltransferase CARM1-like [Source:NCBI gene (formerly Entrezgene);Acc:106509183]
100	ENSSSCG00000028190	Up	HOMEZ	homeobox and leucine zipper encoding [Source:VGNC Symbol;Acc:VGNC:88930]
101	ENSSSCG00000031503	Up	PRRX1	paired-related homeobox 1 [Source:VGNC Symbol;Acc:VGNC:91875]
102	ENSSSCG00000039707	Up	RTKN2	rhotekin 2 [Source:VGNC Symbol;Acc:VGNC:92495]
103	ENSSSCG00000034364	Up	SPECC1	sperm antigen with calponin homology and coiled-coil domains 1 [Source:VGNC Symbol;Acc:VGNC:93398]
104	ENSSSCG00000003789	Up	CTH	cystathionine gamma-lyase [Source:VGNC Symbol;Acc:VGNC:96961]
105	ENSSSCG00000000997	Up	PPP1R3G	protein phosphatase 1 regulatory subunit 3G [Source:VGNC Symbol;Acc:VGNC:91740]
106	ENSSSCG00000008176	Up	LONRF2	LON peptidase N-terminal domain and ring finger 2 [Source:VGNC Symbol;Acc:VGNC:89777]
107	ENSSSCG00000014437	Up	PPARGC1B	PPARG coactivator 1 beta [Source:VGNC Symbol;Acc:VGNC:91686]
108	ENSSSCG00000017585	Up	SAMD14	sterile alpha motif domain-containing 14 [Source:VGNC Symbol;Acc:VGNC:92566]
109	ENSSSCG00000006932	Up	CLCA4	calcium-activated chloride channel regulator 4-like [Source:NCBI gene (formerly Entrezgene);Acc:100512780]
110	ENSSSCG00000031616	Up	FOSB	FosB proto-oncogene, AP-1 transcription factor subunit [Source:VGNC Symbol;Acc:VGNC:88190]
111	ENSSSCG00000000419	Up	RDH16	retinol dehydrogenase 16 [Source:NCBI gene (formerly Entrezgene);Acc:100626199]
112	ENSSSCG00000032557	Up	LDHB	lactate dehydrogenase B [Source:NCBI gene (formerly Entrezgene);Acc:100621540]
113	ENSSSCG00000010640	Up	NRAP	nebulin-related anchoring protein [Source:VGNC Symbol;Acc:VGNC:90888]
114	ENSSSCG00000002383	Up	FOS	Fos proto-oncogene, AP-1 transcription factor subunit [Source:NCBI gene (formerly Entrezgene);Acc:100144486]
115	ENSSSCG00000011264	Up	CSRNP1	cysteine- and serine-rich nuclear protein 1 [Source:VGNC Symbol;Acc:VGNC:87046]
116	ENSSSCG00000004392	Up	AMD1	adenosylmethionine decarboxylase 1 [Source:NCBI gene (formerly Entrezgene);Acc:100155925]
117	ENSSSCG00000005475	Up	-	ZFP37 zinc finger protein [Source:NCBI gene (formerly Entrezgene);Acc:100517074]
118	ENSSSCG00000002427	Up	EML5	EMAP-like 5 [Source:VGNC Symbol;Acc:VGNC:87686]
119	ENSSSCG00000012050	Up	RCAN1	regulator of calcineurin 1 [Source:VGNC Symbol;Acc:VGNC:92170]

120	ENSSSCG00000026259	Up	LINGO4	leucine-rich repeat- and Ig domain-containing 4 [Source:VGNC Symbol;Acc:VGNC:89735]
121	ENSSSCG00000003928	Up	PLK3	polo-like kinase 3 [Source:VGNC Symbol;Acc:VGNC:91564]
122	ENSSSCG00000010926	Up	SYT2	synaptotagmin 2 [Source:VGNC Symbol;Acc:VGNC:95530]
123	ENSSSCG00000005086	Up	SIX6	SIX homeobox 6 [Source:VGNC Symbol;Acc:VGNC:92897]
124	ENSSSCG00000032715	Up	CERS6	ceramide synthase 6 [Source:VGNC Symbol;Acc:VGNC:96025]
125	ENSSSCG00000002029	Up	MYH7	myosin heavy chain 7 [Source:NCBI gene (formerly Entrezgene);Acc:396860]
126	ENSSSCG00000041777	Up	-	-
127	ENSSSCG00000034410	Up	TBX10	T-box transcription factor 10 [Source:HGNC Symbol;Acc:HGNC:11593]
128	ENSSSCG00000023791	Up	-	-
129	ENSSSCG00000001202	Up	-	-
130	ENSSSCG00000035774	Up	ERRFI1	ERBB receptor feedback inhibitor 1 [Source:VGNC Symbol;Acc:VGNC:87784]
131	ENSSSCG00000010894	Up	TP53BP2	tumor protein p53-binding protein 2 [Source:VGNC Symbol;Acc:VGNC:95553]
132	ENSSSCG00000000939	Up	ACSS3	acyl-CoA synthetase short chain family member 3 [Source:VGNC Symbol;Acc:VGNC:85037]
133	ENSSSCG00000011766	Up	PEX5L	peroxisomal biogenesis factor 5-like [Source:VGNC Symbol;Acc:VGNC:91329]
134	ENSSSCG00000025996	Up	-	microtubule-associated scaffold protein 2 [Source:VGNC Symbol;Acc:VGNC:90477]
135	ENSSSCG00000032831	Up	BRI3BP	BRI3-binding protein [Source:VGNC Symbol;Acc:VGNC:85879]
136	ENSSSCG00000009278	Up	FGF9	fibroblast growth factor 9 [Source:NCBI gene (formerly Entrezgene);Acc:396717]
137	ENSSSCG00000037455	Up	NDUFS8	NADH:ubiquinone oxidoreductase core subunit S8 [Source:VGNC Symbol;Acc:VGNC:90656]
138	ENSSSCG00000013303	Up	ABTB2	ankyrin repeat and BTB domain-containing 2 [Source:VGNC Symbol;Acc:VGNC:84994]
139	ENSSSCG00000025483	Up	GREB1	growth-regulating estrogen receptor-binding 1 [Source:VGNC Symbol;Acc:VGNC:96583]
140	ENSSSCG00000004869	Up	CNDP1	carnosine dipeptidase 1 [Source:VGNC Symbol;Acc:VGNC:86811]
141	ENSSSCG00000031307	Up	-	-
142	ENSSSCG00000003230	Up	-	claudin domain-containing 2 [Source:VGNC Symbol;Acc:VGNC:86743]
143	ENSSSCG00000040348	Up	LIN7A	lin-7 homolog A, crumbs cell polarity complex component [Source:VGNC Symbol;Acc:VGNC:89730]
144	ENSSSCG00000036074	Up	AP1S2	adaptor-related protein complex 1 subunit sigma 2 [Source:VGNC Symbol;Acc:VGNC:85382]
145	ENSSSCG00000011168	Up	ZNF510	zinc finger protein 510 [Source:VGNC Symbol;Acc:VGNC:96203]
146	ENSSSCG00000002690	Up	GAN	gigaxonin [Source:VGNC Symbol;Acc:VGNC:88343]

147	ENSSSCG00000001523	Up	GRM4	glutamate metabotropic receptor 4 [Source:VGNC Symbol;Acc:VGNC:98027]
148	ENSSSCG00000034167	Up	SLC5A3	solute carrier family 5 member 3 [Source:VGNC Symbol;Acc:VGNC:93144]
149	ENSSSCG00000009233	Up	GPAT3	glycerol-3-phosphate acyltransferase 3 [Source:VGNC Symbol;Acc:VGNC:88572]
150	ENSSSCG00000030165	Up	MAFF	MAF bZIP transcription factor F [Source:VGNC Symbol;Acc:VGNC:89947]
151	ENSSSCG00000039045	Up	SLC26A2	solute carrier family 26 member 2 [Source:VGNC Symbol;Acc:VGNC:93027]
152	ENSSSCG00000009100	Up	TNIP3	TNFAIP3 interacting protein 3 [Source:VGNC Symbol;Acc:VGNC:94277]
153	ENSSSCG00000045919	Up	-	-
154	ENSSSCG00000023261	Up	GDF5	growth differentiation factor 5 [Source:VGNC Symbol;Acc:VGNC:96319]
155	ENSSSCG00000025503	Up	ADGRG5	adhesion G protein-coupled receptor G5 [Source:VGNC Symbol;Acc:VGNC:85132]
156	ENSSSCG00000039009	Up	-	prune homolog 2 with BCH domain [Source:NCBI gene (formerly Entrezgene);Acc:100153374]
157	ENSSSCG00000038848	Up	IL22RA1	interleukin 22 receptor subunit alpha 1 [Source:VGNC Symbol;Acc:VGNC:89097]
158	ENSSSCG00000032330	Down	THY1	Thy-1 cell surface antigen [Source:VGNC Symbol;Acc:VGNC:93973]
159	ENSSSCG00000037803	Down	MARCKS	myristoylated alanine-rich protein kinase C substrate [Source:VGNC Symbol;Acc:VGNC:90024]
160	ENSSSCG00000016034	Down	COL3A1	collagen type III alpha 1 chain [Source:VGNC Symbol;Acc:VGNC:95979]
161	ENSSSCG00000000138	Down	PVALB	parvalbumin [Source:VGNC Symbol;Acc:VGNC:92007]
162	ENSSSCG00000008617	Down	CYRIA	CYFIP-related Rac1 interactor A [Source:VGNC Symbol;Acc:VGNC:87131]
163	ENSSSCG00000015522	Down	ANGPTL1	angiopoietin-like 1 [Source:VGNC Symbol;Acc:VGNC:85303]
164	ENSSSCG00000016174	Down	FN1	fibronectin 1 [Source:VGNC Symbol;Acc:VGNC:96305]
165	ENSSSCG00000000916	Down	LUM	lumican [Source:VGNC Symbol;Acc:VGNC:89894]
166	ENSSSCG00000035937	Down	DPT	dermatopontin [Source:VGNC Symbol;Acc:VGNC:98784]
167	ENSSSCG00000036135	Down	COL1A1	collagen type I alpha 1 chain [Source:VGNC Symbol;Acc:VGNC:86870]
168	ENSSSCG00000040513	Down	AQP3	aquaporin 3 [Source:VGNC Symbol;Acc:VGNC:96671]
169	ENSSSCG00000034720	Down	IQCK	IQ motif-containing K [Source:VGNC Symbol;Acc:VGNC:89191]
170	ENSSSCG00000000915	Down	DCN	decorin [Source:NCBI gene (formerly Entrezgene);Acc:396957]
171	ENSSSCG00000015326	Down	COL1A2	collagen type I alpha 2 chain [Source:VGNC Symbol;Acc:VGNC:86871]
172	ENSSSCG00000012576	Down	CHRDL1	chordin-like 1 [Source:VGNC Symbol;Acc:VGNC:86662]
173	ENSSSCG00000037430	Down	COL6A6	collagen type VI alpha 6 chain [Source:NCBI gene (formerly Entrezgene);Acc:100516642]
174	ENSSSCG00000005997	Down	COL14A1	collagen type XIV alpha 1 chain [Source:VGNC Symbol;Acc:VGNC:97936]

175	ENSSSCG00000040162	Down	NUPR1	nuclear protein 1, transcriptional regulator [Source:VGNC Symbol;Acc:VGNC:90989]
176	ENSSSCG00000011522	Down	CNTN3	contactin 3 [Source:NCBI gene (formerly Entrezgene);Acc:100511594]
177	ENSSSCG00000034607	Down	OGN	osteoglycin [Source:VGNC Symbol;Acc:VGNC:91028]
178	ENSSSCG00000001478	Down	BMP5	bone morphogenetic protein 5 [Source:VGNC Symbol;Acc:VGNC:85844]
179	ENSSSCG00000037697	Down	MGP	matrix Gla protein [Source:VGNC Symbol;Acc:VGNC:90201]
180	ENSSSCG00000015662	Down	C4BPA	complement component 4-binding protein, alpha [Source:NCBI gene (formerly Entrezgene);Acc:396982]
181	ENSSSCG00000009672	Down	-	scavenger receptor class A member 5 [Source:VGNC Symbol;Acc:VGNC:92612]
182	ENSSSCG00000004658	Down	FBN1	fibrillin 1 [Source:NCBI gene (formerly Entrezgene);Acc:414836]
183	ENSSSCG00000024043	Down	ADAMTS2	ADAM metallopeptidase with thrombospondin type 1 motif 2 [Source:VGNC Symbol;Acc:VGNC:85081]
184	ENSSSCG00000037534	Down	OPCML	opioid-binding protein/cell adhesion molecule-like [Source:VGNC Symbol;Acc:VGNC:91047]
185	ENSSSCG00000023924	Down	PDE1A	phosphodiesterase 1A [Source:VGNC Symbol;Acc:VGNC:96473]
186	ENSSSCG00000039415	Down	CCDC197	coiled-coil domain-containing 197 [Source:HGNC Symbol;Acc:HGNC:19860]
187	ENSSSCG00000016035	Down	COL5A2	collagen type V alpha 2 chain [Source:VGNC Symbol;Acc:VGNC:95981]
188	ENSSSCG00000005751	Down	COL5A1	collagen type V alpha 1 chain [Source:VGNC Symbol;Acc:VGNC:86877]
189	ENSSSCG00000031538	Down	RNASE4	angiogenin [Source:NCBI gene (formerly Entrezgene);Acc:733639]
190	ENSSSCG00000017534	Down	HOXB3	homeobox B3 [Source:VGNC Symbol;Acc:VGNC:88944]
191	ENSSSCG00000021557	Down	SULT1A3	sulfotransferase family 1A member 3 [Source:NCBI gene (formerly Entrezgene);Acc:396640]
192	ENSSSCG00000011928	Down	CCDC80	coiled-coil domain-containing 80 [Source:VGNC Symbol;Acc:VGNC:86315]
193	ENSSSCG00000009699	Down	HPGD	15-hydroxyprostaglandin dehydrogenase [Source:VGNC Symbol;Acc:VGNC:88961]
194	ENSSSCG00000032241	Down	GPNMB	glycoprotein nmb [Source:VGNC Symbol;Acc:VGNC:88593]
195	ENSSSCG00000000887	Down	-	lamina-associated polypeptide 2, isoforms beta/gamma [Source:NCBI gene (formerly Entrezgene);Acc:100622780]
196	ENSSSCG00000017300	Down	MRC2	mannose receptor C type 2 [Source:VGNC Symbol;Acc:VGNC:90341]
197	ENSSSCG00000015896	Down	FAP	fibroblast activation protein alpha [Source:VGNC Symbol;Acc:VGNC:99714]
198	ENSSSCG00000016032	Down	TFPI	tissue factor pathway inhibitor [Source:VGNC Symbol;Acc:VGNC:98366]
199	ENSSSCG00000004052	Down	FNDC1	fibronectin type III domain-containing 1 [Source:VGNC Symbol;Acc:VGNC:88181]

200	ENSSSCG00000028549	Down	ECM2	extracellular matrix protein 2 [Source:VGNC Symbol;Acc:VGNC:87534]
201	ENSSSCG00000004291	Down	NT5E	5'-nucleotidase ecto [Source:VGNC Symbol;Acc:VGNC:90925]
202	ENSSSCG00000026710	Down	CARHSP1	calcium-regulated heat stable protein 1 [Source:VGNC Symbol;Acc:VGNC:86190]
203	ENSSSCG00000012132	Down	ASB9	ankyrin repeat- and SOCS box-containing 9 [Source:VGNC Symbol;Acc:VGNC:85568]
204	ENSSSCG00000011683	Down	PAQR9	procollagen C-endopeptidase enhancer 2 [Source:NCBI gene (formerly Entrezgene);Acc:100156529]
205	ENSSSCG00000036452	Down	LRRC17	leucine-rich repeat-containing 17 [Source:VGNC Symbol;Acc:VGNC:89828]
206	ENSSSCG00000005455	Down	SVEP1	sushi, von Willebrand factor type A, EGF and pentraxin domain-containing 1 [Source:VGNC Symbol;Acc:VGNC:93642]
207	ENSSSCG00000008841	Down	PDGFRA	Platelet-derived growth factor receptor alpha [Source:VGNC Symbol;Acc:VGNC:98179]
208	ENSSSCG00000017082	Down	SPARC	secreted protein acidic and cysteine-rich [Source:VGNC Symbol;Acc:VGNC:98332]
209	ENSSSCG00000035074	Down	FOXO6	forkhead box O6 [Source:NCBI gene (formerly Entrezgene);Acc:110261252]
210	ENSSSCG00000015037	Down	IL18	interleukin 18 [Source:NCBI gene (formerly Entrezgene);Acc:397057]
211	ENSSSCG00000046261	Down	TEX26	testis-expressed 26 [Source:NCBI gene (formerly Entrezgene);Acc:100516873]
212	ENSSSCG00000015322	Down	TFPI2	tissue factor pathway inhibitor 2 [Source:VGNC Symbol;Acc:VGNC:93922]
213	ENSSSCG00000048323	Down	-	Xg glycoprotein (Xg blood group) [Source:NCBI gene (formerly Entrezgene);Acc:102166402]
214	ENSSSCG00000009526	Down	ITGBL1	integrin subunit beta-like 1 [Source:VGNC Symbol;Acc:VGNC:89247]
215	ENSSSCG00000007235	Down	TPX2	TPX2 microtubule nucleation factor [Source:VGNC Symbol;Acc:VGNC:95559]
216	ENSSSCG00000008606	Down	OSR1	odd-skipped-related transcription factor 1 [Source:VGNC Symbol;Acc:VGNC:91083]
217	ENSSSCG00000023611	Down	-	tenascin XB [Source:NCBI gene (formerly Entrezgene);Acc:445520]
218	ENSSSCG00000013901	Down	IFI30	IFI30 lysosomal thiol reductase [Source:VGNC Symbol;Acc:VGNC:89031]
219	ENSSSCG00000027466	Down	PCOLCE	procollagen C-endopeptidase enhancer [Source:VGNC Symbol;Acc:VGNC:91227]
220	ENSSSCG00000037508	Down	GSN	gelsolin [Source:HGNC Symbol;Acc:HGNC:4620]
221	ENSSSCG00000012482	Down	SRPX2	sushi repeat-containing protein X-linked 2 [Source:NCBI gene (formerly Entrezgene);Acc:100153130]
222	ENSSSCG00000008898	Down	-	HOP homeobox [Source:VGNC Symbol;Acc:VGNC:88932]
223	ENSSSCG00000009489	Down	GPC6	glypican 6 [Source:NCBI gene (formerly Entrezgene);Acc:102159116]
224	ENSSSCG00000002444	Down	FBLN5	fibulin 5 [Source:VGNC Symbol;Acc:VGNC:88024]

225	ENSSSCG00000028185	Down	FGD3	FYVE, RhoGEF and PH domain-containing 3 [Source:VGNC Symbol;Acc:VGNC:88096]
226	ENSSSCG00000012911	Down	CARNS1	carnosine synthase 1 [Source:VGNC Symbol;Acc:VGNC:97913]
227	ENSSSCG00000016816	Down	ADAMTS12	ADAM metallopeptidase with thrombospondin type 1 motif 12 [Source:VGNC Symbol;Acc:VGNC:85074]
228	ENSSSCG00000005785	Down	PCSK6	proprotein convertase subtilisin/kexin type 6 [Source:NCBI gene (formerly Entrezgene);Acc:100524545]
229	ENSSSCG00000008397	Down	EFEMP1	EGF-containing fibulin extracellular matrix protein 1 [Source:NCBI gene (formerly Entrezgene);Acc:100512046]
230	ENSSSCG00000031712	Down	MFAP5	microfibril-associated protein 5 [Source:VGNC Symbol;Acc:VGNC:90177]
231	ENSSSCG00000037015	Down	SESN3	sestrin 3 [Source:VGNC Symbol;Acc:VGNC:92755]
232	ENSSSCG00000040607	Down	MAF	MAF bZIP transcription factor [Source:VGNC Symbol;Acc:VGNC:89945]
233	ENSSSCG00000009671	Down	PBK	PDZ-binding kinase [Source:VGNC Symbol;Acc:VGNC:91200]
234	ENSSSCG00000017511	Down	PLXDC1	plexin domain-containing 1 [Source:VGNC Symbol;Acc:VGNC:91578]
235	ENSSSCG00000015045	Down	NCAM1	neural cell adhesion molecule 1 [Source:NCBI gene (formerly Entrezgene);Acc:100515564]
236	ENSSSCG00000012741	Down	MAMLD1	mastermind-like domain-containing 1 [Source:NCBI gene (formerly Entrezgene);Acc:100517073]
237	ENSSSCG00000021205	Down	STK26	serine/threonine kinase 26 [Source:VGNC Symbol;Acc:VGNC:98346]
238	ENSSSCG00000034866	Down	-	transforming acidic coiled-coil-containing protein 1 [Source:NCBI gene (formerly Entrezgene);Acc:106506226]
239	ENSSSCG00000009004	Down	SFRP2	secreted frizzled-related protein 2 [Source:VGNC Symbol;Acc:VGNC:92775]
240	ENSSSCG00000005650	Down	CERCAM	cerebral endothelial cell adhesion molecule [Source:VGNC Symbol;Acc:VGNC:86585]
241	ENSSSCG00000016943	Down	ADAMTS6	ADAM metallopeptidase with thrombospondin type 1 motif 6 [Source:VGNC Symbol;Acc:VGNC:85086]
242	ENSSSCG00000010655	Down	GFRA1	GDNF family receptor alpha 1 [Source:VGNC Symbol;Acc:VGNC:88428]
243	ENSSSCG00000007748	Down	PSPH	phosphoserine phosphatase [Source:VGNC Symbol;Acc:VGNC:91931]
244	ENSSSCG00000016665	Down	BMPER	BMP-binding endothelial regulator [Source:VGNC Symbol;Acc:VGNC:97902]
245	ENSSSCG00000008491	Down	QPCT	glutaminyl-peptide cyclotransferase [Source:VGNC Symbol;Acc:VGNC:92026]
246	ENSSSCG00000004241	Down	GJA1	gap junction protein alpha 1 [Source:NCBI gene (formerly Entrezgene);Acc:100518636]
247	ENSSSCG00000006345	Down	OLFML2B	olfactomedin-like 2B [Source:VGNC Symbol;Acc:VGNC:91034]

248	ENSSSCG00000009745	Down	ADGRD1	adhesion G protein-coupled receptor D1 [Source:NCBI gene (formerly Entrezgene);Acc:100736874]
249	ENSSSCG00000000607	Down	ART4	ADP-ribosyltransferase 4 (Dombrock blood group) [Source:NCBI gene (formerly Entrezgene);Acc:100152653]
250	ENSSSCG00000015271	Down	PRELP	proline- and arginine-rich end leucine-rich repeat protein [Source:VGNC Symbol;Acc:VGNC:91789]
251	ENSSSCG00000002921	Down	CLIP3	CAP-Gly domain-containing linker protein 3 [Source:VGNC Symbol;Acc:VGNC:97931]
252	ENSSSCG00000022868	Down	KCTD4	potassium channel tetramerization domain-containing 4 [Source:VGNC Symbol;Acc:VGNC:89401]
253	ENSSSCG00000024396	Down	NOVA1	NOVA alternative splicing regulator 1 [Source:VGNC Symbol;Acc:VGNC:90827]
254	ENSSSCG00000017256	Down	ABCA6	ATP-binding cassette subfamily A member 6 [Source:VGNC Symbol;Acc:VGNC:84950]
255	ENSSSCG00000004149	Down	NHSL1	NHS-like 1 [Source:VGNC Symbol;Acc:VGNC:90739]
256	ENSSSCG00000002039	Down	MMP14	matrix metallopeptidase 14 [Source:NCBI gene (formerly Entrezgene);Acc:397471]
257	ENSSSCG00000006735	Down	PTGFRN	prostaglandin F2 receptor inhibitor [Source:VGNC Symbol;Acc:VGNC:91955]
258	ENSSSCG00000033727	Down	GPX1	glutathione peroxidase 1 [Source:VGNC Symbol;Acc:VGNC:88652]
259	ENSSSCG00000030325	Down	C1QTNF6	C1q- and TNF-related 6 [Source:VGNC Symbol;Acc:VGNC:85985]
260	ENSSSCG00000000105	Down	KDELR3	KDEL endoplasmic reticulum protein retention receptor 3 [Source:VGNC Symbol;Acc:VGNC:89406]
261	ENSSSCG00000026180	Down	NRXN1	neurexin 1 [Source:NCBI gene (formerly Entrezgene);Acc:100519087]
262	ENSSSCG00000025686	Down	KMO	kynurenine 3-monooxygenase [Source:VGNC Symbol;Acc:VGNC:96398]
263	ENSSSCG00000033919	Down	DCLK1	doublecortin-like kinase 1 [Source:NCBI gene (formerly Entrezgene);Acc:100623824]
264	ENSSSCG00000038540	Down	-	olfactomedin-like 1 [Source:NCBI gene (formerly Entrezgene);Acc:100622122]
265	ENSSSCG00000006082	Down	MATN2	matrilin 2 [Source:VGNC Symbol;Acc:VGNC:90043]
266	ENSSSCG00000036383	Down	LGALS3BP	galectin 3-binding protein [Source:VGNC Symbol;Acc:VGNC:89695]
267	ENSSSCG00000024403	Down	PRRT1	proline-rich transmembrane protein 1 [Source:VGNC Symbol;Acc:VGNC:91871]
268	ENSSSCG00000025834	Down	FNDC5	fibronectin type III domain-containing 5 [Source:VGNC Symbol;Acc:VGNC:88185]
269	ENSSSCG00000035058	Down	PID1	phosphotyrosine interaction domain-containing 1 [Source:VGNC Symbol;Acc:VGNC:96114]
270	ENSSSCG00000028135	Down	PRTFDC1	phosphoribosyl transferase domain-containing 1 [Source:VGNC Symbol;Acc:VGNC:95996]

271	ENSSSCG00000040629	Down	IL34	interleukin 34 [Source:VGNC Symbol;Acc:VGNC:89107]
272	ENSSSCG00000012234	Down	SRPX	sushi repeat-containing protein X-linked [Source:NCBI gene (formerly Entrezgene);Acc:100156108]
273	ENSSSCG00000006087	Down	CPQ	carboxypeptidase Q [Source:VGNC Symbol;Acc:VGNC:86958]
274	ENSSSCG00000008785	Down	FAM114A1	family with sequence similarity 114 member A1 [Source:VGNC Symbol;Acc:VGNC:87890]
275	ENSSSCG00000040566	Down	N4BP2L1	NEDD4-binding protein 2-like 1 [Source:NCBI gene (formerly Entrezgene);Acc:100153367]
276	ENSSSCG00000031514	Down	CCDC28B	coiled-coil domain-containing 28B [Source:VGNC Symbol;Acc:VGNC:86290]
277	ENSSSCG00000007385	Down	KCNS1	potassium voltage-gated channel modifier subfamily S member 1 [Source:VGNC Symbol;Acc:VGNC:96391]
278	ENSSSCG00000027726	Down	DLL1	delta-like canonical Notch ligand 1 [Source:VGNC Symbol;Acc:VGNC:87335]
279	ENSSSCG00000006578	Down	S100A4	S100 calcium-binding protein A4 [Source:VGNC Symbol;Acc:VGNC:92543]
280	ENSSSCG00000005446	Down	EPB41L4B	erythrocyte membrane protein band 4.1-like 4B [Source:NCBI gene (formerly Entrezgene);Acc:100510946]
281	ENSSSCG00000012001	Down	ROBO1	roundabout guidance receptor 1 [Source:NCBI gene (formerly Entrezgene);Acc:100517310]
282	ENSSSCG00000006001	Down	ENPP2	ectonucleotide pyrophosphatase/phosphodiesterase 2 [Source:VGNC Symbol;Acc:VGNC:87709]
283	ENSSSCG00000023328	Down	GPM6A	glycoprotein M6A [Source:VGNC Symbol;Acc:VGNC:98025]
284	ENSSSCG00000012546	Down	NRK	Nik-related kinase [Source:VGNC Symbol;Acc:VGNC:98161]
285	ENSSSCG00000031640	Down	-	endogenous retrovirus group V member 2 Env polyprotein [Source:NCBI gene (formerly Entrezgene);Acc:100624077]
286	ENSSSCG00000002341	Down	PAPLN	papilin, proteoglycan-like sulfated glycoprotein [Source:VGNC Symbol;Acc:VGNC:91169]
287	ENSSSCG00000007170	Down	CPXM1	carboxypeptidase X, M14 family member 1 [Source:VGNC Symbol;Acc:VGNC:95659]
288	ENSSSCG00000002620	Down	EFHC1	EF-hand domain-containing 1 [Source:VGNC Symbol;Acc:VGNC:87570]
289	ENSSSCG00000002632	Down	SLC28A1	solute carrier family 28 member 1 [Source:VGNC Symbol;Acc:VGNC:93041]
290	ENSSSCG00000040089	Down	TEDC1	tubulin epsilon and delta complex 1 [Source:VGNC Symbol;Acc:VGNC:93861]
291	ENSSSCG00000017066	Down	GEMIN5	gem nuclear organelle-associated protein 5 [Source:VGNC Symbol;Acc:VGNC:88413]
292	ENSSSCG00000000277	Down	NPFF	neuropeptide FF-amide peptide precursor [Source:VGNC Symbol;Acc:VGNC:90845]
293	ENSSSCG00000011216	Down	LRRC3B	leucine-rich repeat-containing 3B [Source:VGNC Symbol;Acc:VGNC:89838]
294	ENSSSCG00000012773	Down	PNCK	pregnancy up-regulated nonubiquitous CaM kinase [Source:VGNC Symbol;Acc:VGNC:91593]

295	ENSSSCG00000040366	Down	ADAMTSL1	ADAMTS-like 1 [Source:VGNC Symbol;Acc:VGNC:85090]
296	ENSSSCG00000026386	Down	SLC30A2	solute carrier family 30 member 2 [Source:VGNC Symbol;Acc:VGNC:93056]
297	ENSSSCG00000021328	Down	PCSK1N	proprotein convertase subtilisin/kexin type 1 inhibitor [Source:VGNC Symbol;Acc:VGNC:91231]
298	ENSSSCG00000011973	Down	COL8A1	collagen type VIII alpha 1 chain [Source:VGNC Symbol;Acc:VGNC:86880]
299	ENSSSCG00000032946	Down	-	high mobility group nucleosome binding domain 5 [Source:NCBI gene (formerly Entrezgene);Acc:100623249]
300	ENSSSCG00000016290	Down	EFHD1	EF-hand domain family member D1 [Source:VGNC Symbol;Acc:VGNC:96222]
301	ENSSSCG00000047413	Down	OTOS	otospiralin [Source:NCBI gene (formerly Entrezgene);Acc:100525436]
302	ENSSSCG00000017873	Down	CAMKK1	calcium/calmodulin-dependent protein kinase kinase 1 [Source:VGNC Symbol;Acc:VGNC:98977]
303	ENSSSCG00000001910	Down	ISLR	immunoglobulin superfamily-containing leucine-rich repeat [Source:VGNC Symbol;Acc:VGNC:98052]
304	ENSSSCG00000025085	Down	NEGR1	neuronal growth regulator 1 [Source:VGNC Symbol;Acc:VGNC:90671]
305	ENSSSCG00000014943	Down	DEUP1	deuterosome assembly protein 1 [Source:VGNC Symbol;Acc:VGNC:87264]
306	ENSSSCG00000041289	Down	IL10RB	interleukin 10 receptor subunit beta [Source:NCBI gene (formerly Entrezgene);Acc:396657]
307	ENSSSCG00000014812	Down	-	folate receptor 2 (fetal) [Source:NCBI gene (formerly Entrezgene);Acc:396853]
308	ENSSSCG00000028981	Down	ZNF367	zinc finger protein 367 [Source:VGNC Symbol;Acc:VGNC:95882]
309	ENSSSCG00000039426	Down	FSTL1	follistatin-like 1 [Source:VGNC Symbol;Acc:VGNC:88255]
310	ENSSSCG00000034570	Down	IFI6	interferon alpha inducible protein 6 [Source:VGNC Symbol;Acc:VGNC:89033]
311	ENSSSCG00000017109	Down	ADAMTS16	ADAM metallopeptidase with thrombospondin type 1 motif 16 [Source:VGNC Symbol;Acc:VGNC:85078]
312	ENSSSCG00000010584	Down	MFSD13A	major facilitator superfamily domain-containing 13A [Source:VGNC Symbol;Acc:VGNC:90186]
313	ENSSSCG00000036307	Down	OAF	out at first homolog [Source:VGNC Symbol;Acc:VGNC:91005]
314	ENSSSCG00000048752	Down	-	-
315	ENSSSCG00000045531	Down	FKBP1B	FKBP prolyl isomerase 1B [Source:NCBI gene (formerly Entrezgene);Acc:100522131]
316	ENSSSCG00000015999	Down	FKBP7	FKBP prolyl isomerase 7 [Source:VGNC Symbol;Acc:VGNC:96302]
317	ENSSSCG00000017538	Down	HOXB7	homeobox B7 [Source:VGNC Symbol;Acc:VGNC:88947]
318	ENSSSCG00000003399	Down	RBP7	retinol-binding protein 7 [Source:VGNC Symbol;Acc:VGNC:92165]

319	ENSSSCG00000024476	Down	CES3	carboxylesterase 3 [Source:VGNC Symbol;Acc:VGNC:96948]
320	ENSSSCG00000014436	Down	ARHGEF37	Rho guanine nucleotide exchange factor 37 [Source:VGNC Symbol;Acc:VGNC:85498]
321	ENSSSCG00000015175	Down	VWA5A	von Willebrand factor A domain-containing 5A [Source:VGNC Symbol;Acc:VGNC:94884]
322	ENSSSCG00000016522	Down	PTN	pleiotrophin [Source:VGNC Symbol;Acc:VGNC:91965]
323	ENSSSCG00000002464	Down	-	proline-rich membrane anchor 1 [Source:NCBI gene (formerly Entrezgene);Acc:102167492]
324	ENSSSCG00000029039	Down	-	BRCA2 DNA repair-associated [Source:NCBI gene (formerly Entrezgene);Acc:100624979]
325	ENSSSCG00000011670	Down	PXYLP1	2-phosphoxylose phosphatase 1 [Source:VGNC Symbol;Acc:VGNC:92017]
326	ENSSSCG00000035417	Down	TMEM238	transmembrane protein 238 [Source:VGNC Symbol;Acc:VGNC:94149]
327	ENSSSCG00000024736	Down	ACP4	acid phosphatase 4 [Source:VGNC Symbol;Acc:VGNC:85026]
328	ENSSSCG00000029073	Down	CACNB4	calcium voltage-gated channel auxiliary subunit beta 4 [Source:NCBI gene (formerly Entrezgene);Acc:396585]
329	ENSSSCG00000001931	Down	-	GRAM domain-containing 2A [Source:NCBI gene (formerly Entrezgene);Acc:100158151]
330	ENSSSCG00000000653	Down	CD69	CD69 molecule [Source:NCBI gene (formerly Entrezgene);Acc:397165]
331	ENSSSCG00000049361	Down	-	-
332	ENSSSCG00000002866	Down	CEBPA	CCAAT enhancer-binding protein alpha [Source:VGNC Symbol;Acc:VGNC:86531]
333	ENSSSCG00000004013	Down	SMOC2	SPARC-related modular calcium binding 2 [Source:VGNC Symbol;Acc:VGNC:93260]
334	ENSSSCG00000000854	Down	-	stabilin 2 [Source:NCBI gene (formerly Entrezgene);Acc:100521556]
335	ENSSSCG00000005530	Down	LHX6	LIM homeobox 6 [Source:VGNC Symbol;Acc:VGNC:89716]
336	ENSSSCG00000006140	Down	CA2	carbonic anhydrase 2 [Source:VGNC Symbol;Acc:VGNC:98746]
337	ENSSSCG00000037600	Down	C1orf216	chromosome 6 C1orf216 homolog [Source:VGNC Symbol;Acc:VGNC:86064]
338	ENSSSCG00000003521	Down	-	Wnt family member 4 [Source:VGNC Symbol;Acc:VGNC:94972]
339	ENSSSCG00000006171	Down	CRISPLD1	cysteine-rich secretory protein LCCL domain-containing 1 [Source:VGNC Symbol;Acc:VGNC:97945]
340	ENSSSCG00000020988	Down	-	zinc finger protein 114 [Source:VGNC Symbol;Acc:VGNC:98749]
341	ENSSSCG00000009011	Down	FHDC1	FH2 domain-containing 1 [Source:VGNC Symbol;Acc:VGNC:88126]
342	ENSSSCG00000022728	Down	HOXC10	homeobox C10 [Source:VGNC Symbol;Acc:VGNC:88949]
343	ENSSSCG00000048170	Down	-	-
344	ENSSSCG00000011806	Down	MASP1	Mannan-binding lectin serine peptidase 1 [Source:VGNC Symbol;Acc:VGNC:90032]

345	ENSSSCG00000016516	Down	ATP6V0A4	ATPase H+ transporting V0 subunit a4 [Source:VGNC Symbol;Acc:VGNC:85665]
346	ENSSSCG00000012452	Down	-	SH3 domain-binding glutamate-rich protein-like [Source:VGNC Symbol;Acc:VGNC:92815]
347	ENSSSCG00000032282	Down	ACP5	acid phosphatase 5, tartrate-resistant [Source:VGNC Symbol;Acc:VGNC:85027]
348	ENSSSCG00000003479	Down	MFAP2	microfibril-associated protein 2 [Source:VGNC Symbol;Acc:VGNC:90173]
349	ENSSSCG00000015982	Down	HOXD9	homeobox D9 [Source:VGNC Symbol;Acc:VGNC:96357]
350	ENSSSCG00000003382	Down	TAS1R1	taste 1 receptor member 1 [Source:VGNC Symbol;Acc:VGNC:93740]
351	ENSSSCG00000015019	Down	COLCA2	colorectal cancer-associated 2 [Source:NCBI gene (formerly Entrezgene);Acc:100512618]
352	ENSSSCG00000006051	Down	CTHRC1	collagen triple helix repeat-containing 1 [Source:VGNC Symbol;Acc:VGNC:87061]
353	ENSSSCG00000003881	Down	SPATA6	spermatogenesis-associated 6 [Source:VGNC Symbol;Acc:VGNC:93387]
354	ENSSSCG00000031905	Down	KCNS3	potassium voltage-gated channel modifier subfamily S member 3 [Source:VGNC Symbol;Acc:VGNC:89387]
355	ENSSSCG00000012018	Down	CHODL	chondrolectin [Source:VGNC Symbol;Acc:VGNC:86655]
356	ENSSSCG00000017788	Down	TP53I13	tumor protein p53-inducible protein 13 [Source:VGNC Symbol;Acc:VGNC:94327]
357	ENSSSCG00000001773	Down	TMED3	transmembrane p24 trafficking protein 3 [Source:VGNC Symbol;Acc:VGNC:94055]
358	ENSSSCG00000011412	Down	CACNA2D2	calcium voltage-gated channel auxiliary subunit alpha2delta 2 [Source:VGNC Symbol;Acc:VGNC:86121]
359	ENSSSCG00000011925	Down	CD200R1	CD200 receptor 1 [Source:NCBI gene (formerly Entrezgene);Acc:100155169]
360	ENSSSCG00000035299	Down	SFRP4	secreted frizzled-related protein 4 [Source:VGNC Symbol;Acc:VGNC:92776]
361	ENSSSCG00000013579	Down	CD209	CD209 molecule [Source:NCBI gene (formerly Entrezgene);Acc:100170134]
362	ENSSSCG00000000848	Down	GLT8D2	glycosyltransferase 8 domain-containing 2 [Source:VGNC Symbol;Acc:VGNC:88501]
363	ENSSSCG00000032687	Down	CYP4V2	cytochrome P450, family 4, subfamily v, polypeptide 2 [Source:NCBI gene (formerly Entrezgene);Acc:100113469]
364	ENSSSCG00000011965	Down	TMEM45A	transmembrane protein 45A [Source:VGNC Symbol;Acc:VGNC:94182]
365	ENSSSCG00000010600	Down	CALHM2	calcium homeostasis modulator protein 2 [Source:NCBI gene (formerly Entrezgene);Acc:100157433]
366	ENSSSCG00000016969	Down	-	small EDRK-rich factor 1 [Source:NCBI gene (formerly Entrezgene);Acc:100517502]
367	ENSSSCG00000041846	Down	-	-
368	ENSSSCG00000004961	Down	ITGA11	integrin subunit alpha 11 [Source:VGNC Symbol;Acc:VGNC:89233]

369	ENSSSCG00000034741	Down	HOXD11	homeobox D11 [Source:VGNC Symbol;Acc:VGNC:96352]
370	ENSSSCG00000022337	Down	SERPINB11	serpin family B member 11 [Source:VGNC Symbol;Acc:VGNC:99748]
